# Efficacy and Effectiveness of the Recombinant Zoster Vaccine Against Herpes Zoster, Herpes Zoster Ophthalmicus, Postherpetic Neuralgia and Dementia: A Systematic Review and Meta‐Analysis

**DOI:** 10.1002/rmv.70203

**Published:** 2026-09-16

**Authors:** E. Heen, J. Øverbø, I. H. Tveteraas, S. L. Solberg, H. Nøkleby, J. E. Midtbø, F. Di Rusco, F. Kasteng, A. Ersson, S. Andersson, K. Johansen, L. K. Juvet

**Affiliations:** ^1^ Norwegian Institute of Public Health Oslo Norway; ^2^ Public Health Agency of Sweden Solna Sweden; ^3^ Department of Communicable Disease Control and Prevention Region Jämtland Härjedalen Östersund Sweden; ^4^ Department of Global Public Health Karolinska Institute Stockholm Sweden

## Abstract

Herpes zoster (HZ, shingles) constitutes a significant public health concern, particularly in ageing populations, and there is growing global interest in the recombinant zoster vaccine (RZV) from a public health perspective. In this systematic review with meta‐analysis, we assessed the efficacy and effectiveness of RZV against HZ and its complications, including a possible association with a reduced risk of dementia, among immunocompetent individuals aged 50 years and older. We synthesised the latest evidence on long‐term effects, incorporating recent data from both trials and real‐world effectiveness studies. A total of 27 publications based on 18 primary studies, 3 randomised controlled trials (RCTs) and 15 observational studies, were included. RZV provides very high protection against HZ in immunocompetent adults ≥ 50 years in RCTs, with approximately 95% efficacy and high certainty of evidence (CoE) according to GRADE. Observational studies also consistently indicate substantial protection, but effect estimates vary across settings (high CoE). Among vaccinated individuals who develop HZ, RZV is associated with reduced loss of quality of life and decreased analgesic use. The vaccine also substantially reduces the risk of herpes zoster ophthalmicus (HZO) (high CoE) and likely postherpetic neuralgia (PHN) (moderate CoE). Long‐term follow‐up suggests durable protection for at least 11 years (low CoE). RZV may be associated with a reduced risk of dementia (low CoE), due to uncertainty the true effect may be substantially different, and similar associations have been observed with other vaccines and may be confounded by healthy vaccinee bias.

## 1 Introduction

The World Health Organisation (WHO) highlights life course vaccination strategies towards 2030, and in July 2025, WHO published its *Position Paper on herpes zoster vaccines*, recommending that member states should consider including herpes zoster (HZ, shingles) vaccination in adult immunisation programs to support healthy ageing [1, 2]. Both Sweden and Norway have recently completed national health technology assessments of vaccination against HZ in individuals aged 50 years and older [3, 4].

HZ occurs when the varicella zoster virus (VZV), which causes chickenpox in childhood, is reactivated later in life. After the initial infection, the virus remains dormant in nerve cells. Reactivation of the virus causes nerve inflammation and damage, resulting in pain and a characteristic rash [5, 6]. HZ can also occur after varicella vaccination, but the risk is substantially lower, approximately 80%–90% lower than after natural infection [7, 8]. A decline in immune system function, due to immunoscenescence or immunosuppression caused by disease or therapy, is associated with an increased risk of HZ [5, 6, 9, 10]. This is reflected in the incidence rising with age: individuals over 85 years have approximately twice the risk of those in their early fifties [11], and up to two‐thirds of cases occur in individuals above 50 years [12]. Because varicella infection is common, nearly everyone is at risk of developing HZ. Approximately 30% of individuals will develop HZ during their lifetime, and the overall incidence appears to be increasing with time [11].

HZ usually resolves within 1 month [5, 6, 13]. However, the pain during this period is often severe, difficult to treat, and can substantially reduce quality of life [13, 14, 15, 16]. Up to one‐third of HZ cases develop complications. The most common complication is severe pain lasting at least 90 days after appearance of the rash onset, known as postherpetic neuralgia (PHN), affecting 2%–47% of patients [6, 9, 10, 13, 17]. Other complications are less common but can be serious, including those associated with herpes zoster ophthalmicus (HZO), secondary local bacterial infection, sepsis and neurological complications (e.g., encephalitis and Guillain‐Barré syndrome) [5, 6, 10, 18]. Approximately 10% of individuals with HZ experience a recurrence within the first decade, and up to 25% of these individuals have multiple recurrences [19]. Antiviral treatment reduces the risk of severe disease and complications but requires timely diagnosis and prompt initiation [20].

There is increasing interest in the potential role of VZV in dementia [21, 22]. A plausible hypothesis is that repeated or severe reactivations may contribute to neuroinflammation and vascular damage. VZV reactivation may also indirectly trigger the reactivation of other neurotropic herpesviruses, thereby increasing the risk of dementia [23].

Even aside from the possible association with dementia, HZ and its complications impose a substantial burden on the healthcare system and society [24, 25, 26, 27]. This burden is expected to increase, as populations age and the use of immunosuppressive therapies become more widespread [9, 28].

The only available vaccine, RZV, is an adjuvanted recombinant subunit vaccine (Shingrix, GSK), containing VZV glycoprotein E and the AS01_B_ adjuvant system (GSK). The European Medicines Agency (EMA) granted marketing authorisation for RZV in March 2018. A live attenuated HZ vaccine containing a high dose of VZV virus (ZVL, Zostavax, MSD) was available for prevention of HZ from 2006 until it was discontinued in 2024. In several countries the herpes zoster vaccine is included in the National Immunisation Programs [29], and in many of these countries, the immunisation strategy has shifted from ZVL to RZV [29]. As a result, real‐world studies partly use ZVL as a comparator. Several next‐generation HZ vaccine candidates are currently in clinical development, including mRNA‐based vaccines, recombinant subunit vaccines with other adjuvants, novel antigen‐presenting platforms and an adenovirus vector vaccine [2].

The objective of this systematic review and meta‐analysis is to consolidate the published evidence on the efficacy and effectiveness of RZV against HZ and its complications in immunocompetent persons, including potential protection against dementia. RZV is recommended for risk groups (including immunocompromised individuals) in several countries [29]; these groups are outside the scope of this review.

## 2 Methodology

The systematic review was performed in accordance with the Preferred Reporting Items for Systematic review and Meta‐Analysis (PRISMA) guidelines and was prospectively registered with PROSPERO, CRD42023416345 (https://www.crd.york.ac.uk/PROSPERO/view/CRD42023416345).

A systematic literature search was conducted in the following databases: Embase, MEDLINE (EBSCO), Cochrane Library: Cochrane Database of Systematic Reviews (CDSR), Web of Science and Epistemonikos without restrictions regarding publication language and type. Search strategies were designed by an information specialist and validated by a second information specialist. We developed a conceptually derived search strategy combining “herpes zoster vaccination” and synonyms with “efficacy or effectiveness” and synonyms using Boolean operators. In addition, reference lists of relevant systematic reviews identified in our search were used to identify further relevant studies. The date of the last search was 1 April 2025. Randomized controlled trials (RCTs) and nonrandomised studies of interventions (NRSI) were eligible. Detailed study inclusion/exclusion criteria are reported in Table 1.

**TABLE 1:** PICOS (population, intervention, comparison, outcome, study) for HZ. Inclusion and exclusion criteria.

	Inclusion criteria	Exclusion criteria
Participants	Immunocompetent individuals > 50 years	
Intervention	EU‐approved HZ vaccination available in Norway (recombinant adjuvanted zoster vaccine, RZV, 2 doses)	Studies covering both RZV and ZVL were included, but only RZV‐specific results were extracted and evaluated.
Comparator(s)	Placebo, no vaccine, other vaccines (including live attenuated zoster vaccine; zostavax) or interventions	Studies exclusively assessing ZVL were excluded.
Outcome(s)	Efficacy and effectiveness: Incidence of HZ Incidence of HZ‐ related complications Duration of protection Dementia Cardiovascular disease Mortality	
Study design	Randomized controlled trials (RCTs), and observational studies (NRSIs). *Language:* English, Scandinavian	Case series, narrative reviews, non‐human studies, and immunogenicity studies.

Pairs of reviewers independently screened titles and abstracts of the identified records using Rayyan. At least two independent reviewers assessed the titles and abstracts of all retrieved records. Disagreements were resolved through discussion until consensus was reached. If necessary a third reviewer was consulted to reach a final decision. Data extraction was performed by one researcher and checked against the original study by a second independent reviewer. The following information was extracted from the included studies; study design, setting, duration, funding, follow‐up, sample size, patient characteristics (age, gender), intervention, comparator, relevant outcomes, adjustment factors and a description of the main results. We contacted study authors to request values for any missing data.

At least two reviewers independently assessed the risk of bias and certainty of evidence. For RCTs we used version two of the Cochrane risk‐of‐bias tool 2 for randomized trials (RoB 2) [30] and for observational studies we used version one of the Cochrane Risk Of Bias In Non‐randomized Studies of Interventions (ROBINS‐I) [31].

If multiple publications used substantially overlapping registry populations for the same outcome, we included only one estimate in the primary meta‐analysis to avoid double‐counting participants and over‐weighting a single data source. Selection was based on the most directly relevant comparator for the pooled analysis, length and completeness of follow‐up, adjustment strategy, and publication status.

Vaccine efficacy and effectiveness (VE) were defined as (1−relative effect) × 100, where the relative effect was the risk ratio, rate ratio or hazard ratio reported by the primary studies. The included studies reporting dementia outcomes used different relative association measures, and we treated the dementia meta‐analysis as an approximate synthesis of relative associations rather than an exact pooled risk ratio. Because dementia incidence was below 10% in the included comparative cohorts, we considered this approximation acceptable. When studies reported VE, it was converted to the corresponding relative effect before analysis, using the formula relative effect = 1−VE/100. Pooling was performed on the log scale, and pooled estimates with 95% confidence intervals were backtransformed for presentation. For RCTs, the overall study effect was analysed as an unadjusted incidence‐rate ratio (IRR), derived from the number of confirmed HZ cases and person‐time at risk in the vaccine and placebo groups, with a continuity correction of 0.5 applied for zero cells. For NRSIs, we extracted the maximally adjusted effect estimate reported, log‐transformed the point estimate, and derived standard errors from the reported 95% confidence intervals. Random‐effects restricted maximum likelihood (REML) meta‐analysis was used. For study pools ≥ 5, the truncated Hartung–Knapp–Sidik–Jonkman (HKSJ) adjustment was applied to account for uncertainty in between‐study variance estimation. For study pools < 5, the standard Wald confidence intervals are reported as the primary analysis given the small number of trials. Statistical heterogeneity was assessed using the *I* ^2^ statistic. All analyses were performed in Stata/SE 18 (StataCorp, College Station, TX, USA).

When the 95% CI for the pooled risk ratio did not cross 1, the number needed to vaccinate (NNV) was calculated as the reciprocal of the absolute risk reduction (ARR). For trials reporting person‐years of follow‐up, ARR was estimated as the difference in incidence rates between arms, with standard errors derived from the Poisson variance. The 95% confidence intervals for ARR were computed on the rate‐difference scale and inverted to yield CIs for NNV. Separate analysis was done according to publication type/setting (RCT or NRSI) and outcomes. The certainty of evidence (CoE) was evaluated using the GRADE (Grading of Recommendations Assessment, Development, and Evaluation) approach. Evidence tables were made in GRADEproGDT. In accordance with the GRADE guidelines on rating the certainty of evidence for NRSIs, we started with a high CoE for outcomes assessed with ROBINS‐I [32].

## 3 Results

### 3.1 Study Selection

We identified 21,458 references through databases and hand searches. After removal of duplicates, we screened 12,594 unique references and assessed 136 full text articles. We included a publication released after our search that updated a preregistered study already captured.

In our literature search, we identified six systematic reviews reporting on the effectiveness of RZV [33, 34, 35, 36, 37, 38], and no new primary studies were identified from these reviews.

**FIGURE 1:**
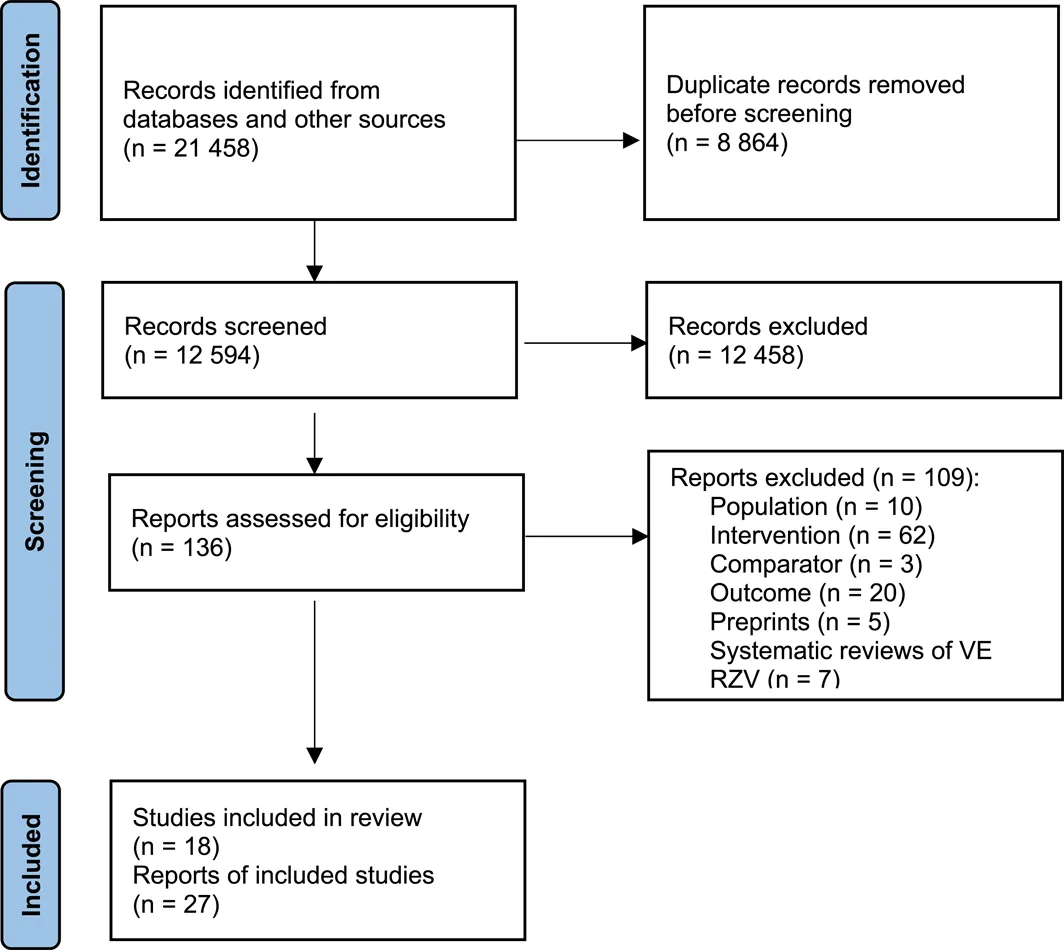
Flow‐chart of identification, screening, exclusion and inclusion of RZV‐studies. Search strategy and the list of excluded studies can be provided by request to corresponding authors.

Finally, we included 27 publications based on 18 primary studies that met the eligibility criteria (Figure 1). An overview of the study design and patient characteristics of the included studies can be found in Table S1.

### 3.2 Study and Patient Characteristics

We included 11 publications based on three RCTs evaluating RZV efficacy against HZ or complications of HZ [39, 40, 41, 42, 43, 44, 45, 46, 47, 48, 49]. Two of the RCTs, ZOE‐50 [39] and ZOE‐70 [40], were phase III trials conducted in 2010–2011 in immunocompetent adults and formed the basis for RZV approval, while the third RCT was a phase IV trial conducted in 2021–2023 [41]. A long‐term follow‐up study (ZOE‐LTFU) followed about half of the vaccinated participants from ZOE‐50 and ZOE‐70 for up to 12 years [42] and used historical controls based on stratified Poisson‐modelled incidence rates. Seven included publications were post‐hoc analyses of pooled data from the ZOE‐trials, reporting VE against additional complications [43], quality of life [44], pain [45] and cardiovascular disease (safety overview) [46], or VE among people with potential immune‐mediated diseases [47], medical conditions [48] and frailty at enrolment [49].

We included 16 publications based on 15 NRSIs evaluating RZV vaccine effectiveness [23, 50, 51, 52, 53, 54, 55, 56, 57, 58, 59, 60, 61, 62, 63, 64]. One of the included publications [58] was released after our search and replaced a preregistered study that had been identified [65].

All NRSIs were conducted in the United States, with up to 5 years of follow‐up. One of the included NRSIs was a case‐control study reported in two publications [56, 57], four were prospective cohort studies [50, 53, 55, 64] and 10 were retrospective cohorts [23, 51, 52, 54, 58, 59, 60, 61, 62, 63]. One study included adults ≥ 18 years (mean age 62–64 years) [56, 57], most included adults ≥ 50 years [50, 52, 54, 55, 58, 59, 60, 61, 62, 63, 64], and three included adults ≥ 65 years [23, 51, 53]. Five studies excluded immunocompromised individuals [54, 59, 60, 61, 62].

### 3.3 Risk of Bias

The risk of bias results are summarised in Table S2. In general, the publications from the primary RCTs had low risk of bias, whereas the long‐term follow‐up study was judged to have high risk of bias due to the use of historical controls. Among the newer NRSIs assessing HZ and PHN, two had moderate risk of bias [50, 64]. Studies examining dementia, mortality, and cardiovascular outcomes had particularly high risk of bias, mainly because of unaddressed confounding factors.

### 3.4 Efficacy and Effectiveness Against HZ

Across the three RCTs, around 17,000 participants received two doses of RZV, with a similar number receiving placebo. Mean age was around 62 years in ZOE‐50 [39] and in Alexandra Echeverria Proano et al. 2024 [41], and around 76 years in ZOE‐70 [40]. Follow‐up was 38 months in ZOE‐50, 43 months in ZOE‐70 and 15 months in Alexandra Echeverria Proano et al. 2024. In the five NRSIs evaluating effectiveness against HZ [50, 53, 59, 60, 64], mean age varied from 61 to 74 years and was unknown in one study [64], average follow‐up was 22 months and more than 2 million vaccinated and 21 million unvaccinated individuals were included. The studies mainly linked vaccination and HZ outcomes using registry data.

**FIGURE 2:**
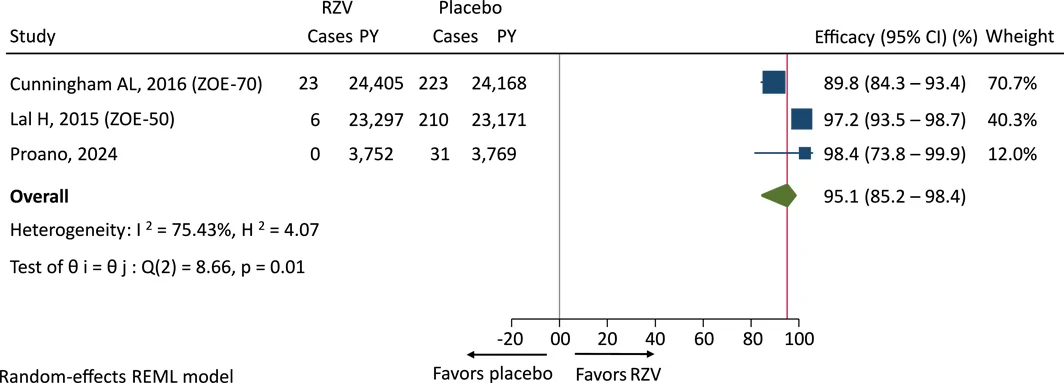
Meta‐analyses VE against HZ from RCTs. Vaccine efficacy was calculated from incidence‐rate ratios based on confirmed HZ cases and person‐years at risk. A continuity correction of 0.5 was applied to Proano et al. because there were no HZ cases in the vaccine arm. PY, person‐years.

**FIGURE 3:**
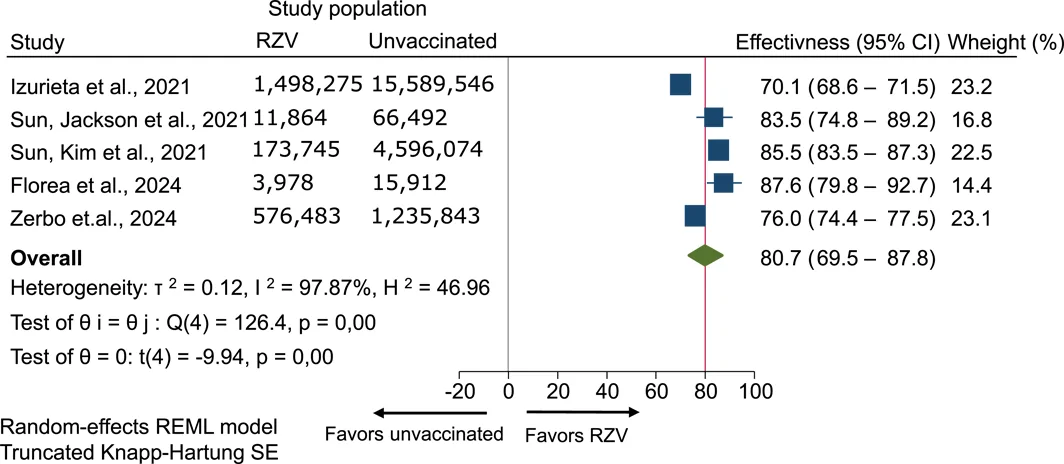
Meta‐analyses of VE against HZ in NRSIs. Estimates are adjusted vaccine‐effectiveness estimates reported by the included non‐randomized studies.

The pooled vaccine efficacy against HZ was 95.1% (95% CI: 85.2–98.4; *I* ^2^ 75.4%) (Figure 2), with consistently high protection across RCTs (high CoE according to GRADE, see Table S3). Based on the randomized trials, 36 individuals ≥ 50 years (NNV 35.5 (95% CI: 31.1–41.3)) and 33 individuals ≥ 70 years (NNV 32.6 (95% CI: 28.3–38.5)) need to be vaccinated to prevent one case of HZ, respectively (over 3.2 and 3.7 years). Meta‐analysis of the NRSIs showed a substantial reduction in HZ risk among vaccinated individuals, but with substantial between‐study heterogeneity. The pooled average VE was 80.7% (95% CI: 69.5–87.8; *I* ^2^ 97.9%) (Figure 3) (high CoE). The 95% prediction interval for the pooled RR was 0.057–0.647, corresponding to a plausible range of true vaccine effectiveness of 35.3%–94.3%, reflecting substantial between‐study heterogeneity. In a sensitivity analysis excluding the two studies that enrolled immunocompromised individuals [53, 64], the estimated VE against HZ was 85.5% (95% CI: 83.6–87.1; *I* ^2^ 0.0%) (data not shown).

**FIGURE 4:**
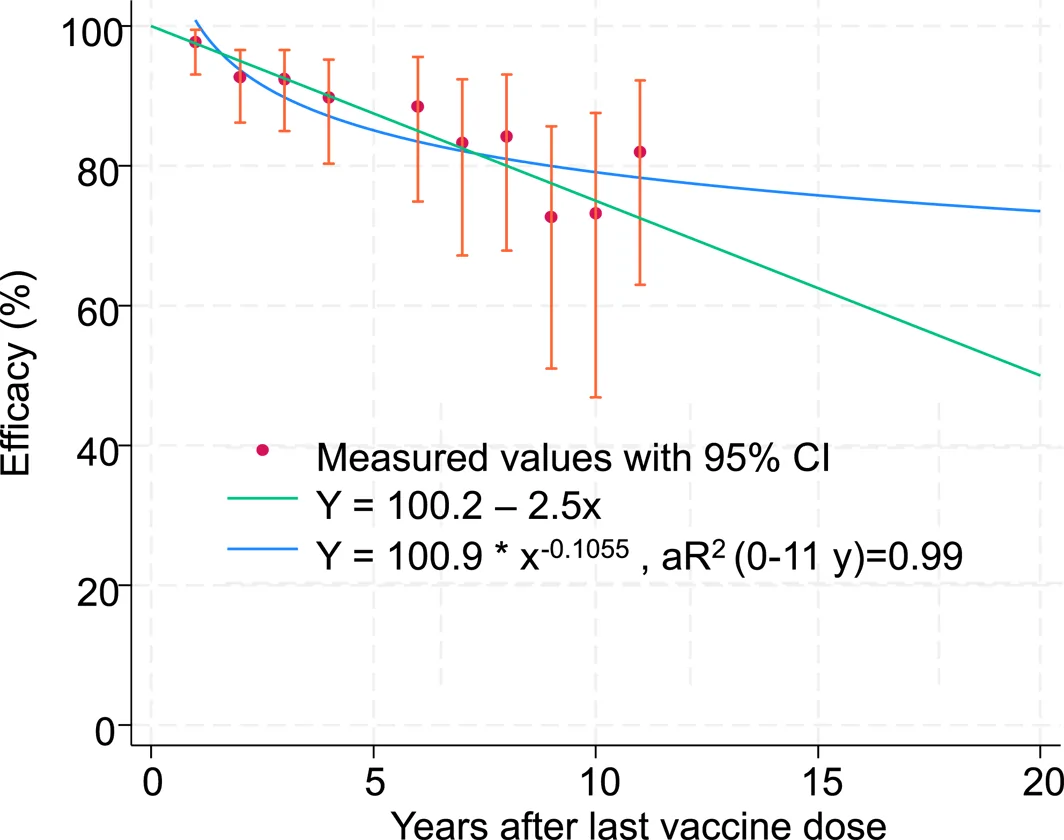
Waning of RZV efficacy against HZ in the ZOE long‐term follow‐up study. Measured annual vaccine‐efficacy estimates with 95% confidence intervals are shown, together with fitted trend lines.

Long‐term follow‐up of the ZOE‐trials showed that RZV provides a substantial reduction in risk of HZ for at least 11 years after vaccination (Figure 4), with less waning in individuals vaccinated before age 70 [42]. VE against HZ from 1 month after dose two until study end was 87.7% (95% CI: 84.9–90.1), and VE against HZ in year 11 remained 82.0% (95% CI: 63.0–92.2) (low CoE). Based on the long‐term follow‐up, 11 individuals ≥ 50 years need to be vaccinated to prevent one case of HZ over 11 years (NNV 11.2 (95% CI: 10.4–12.2)). One NRSI reported VE against HZ at different timepoints until 3.8 years follow‐up [64], VE in year 3 was 73% (95% CI: 68–78).

The RCTs found no significant difference in VE between age groups. However, there was a general trend towards lower VE against HZ with increasing age, both at short and long‐term follow‐up [39, 40, 41, 42] (Table S4). The NRSIs showed a similar pattern [53, 59, 60, 64].

**FIGURE 5:**
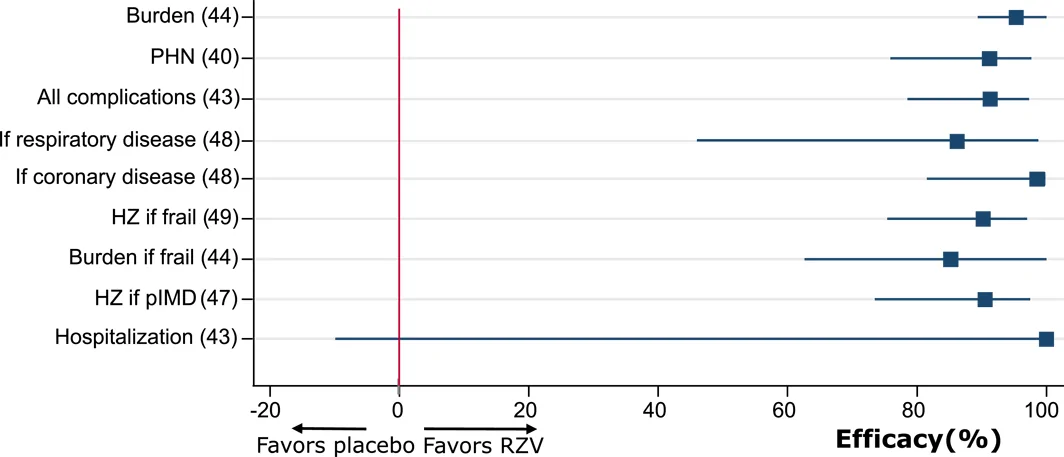
RZV efficacy against selected HZ‐related outcomes in selected subgroups of underlying diseases. Estimates are from post hoc analyses of the ZOE trials. “Burden” refers to HZ burden of illness, reflecting incidence, severity, and duration of HZ‐related symptoms as reported in the source study. HZ, herpes zoster; PHN, postherpetic neuralgia; pIMD, potential immune‐mediated disease.

Post‐hoc analyses of the RCTs showed that VE against HZ was 90.2% (95% CI: 75.4–97.0) in frail individuals [49], 84.5% (95% CI: 46.4–97.1), in those with respiratory disease and 97.0% (95% CI: 82.3–99.9) in those with coronary disease [48]. The vaccine also showed good effect against HZ in individuals with potentially immune‐mediated diseases [47] (Figure 5).

### 3.5 Efficacy and Effectiveness Against PHN

As shown in Figure 5 pooled data from all ZOE participants aged ≥ 50 years showed VE against PHN of 91.2% (95% CI: 75.9–97.7) (moderate CoE) [40]. In the long‐term follow‐up, VE against PHN after 11 years was 87.5% (95% CI: 64.8–96.8) (low CoE) [42]. Two NRSIs with < 1 year of follow‐up assessed PHN [50, 53]. Florea et al. 2024 found no PHN cases among vaccinated individuals (vs. 19 in the unvaccinated group), thus VE against PHN could not be estimated [50]. Izurieta et al. 2021 reported a VE against PHN of 76% (95% CI: 68.4–81.8) (moderate CoE, see Table S3) [53].

### 3.6 Efficacy and Effectiveness Against HZO

**FIGURE 6:**
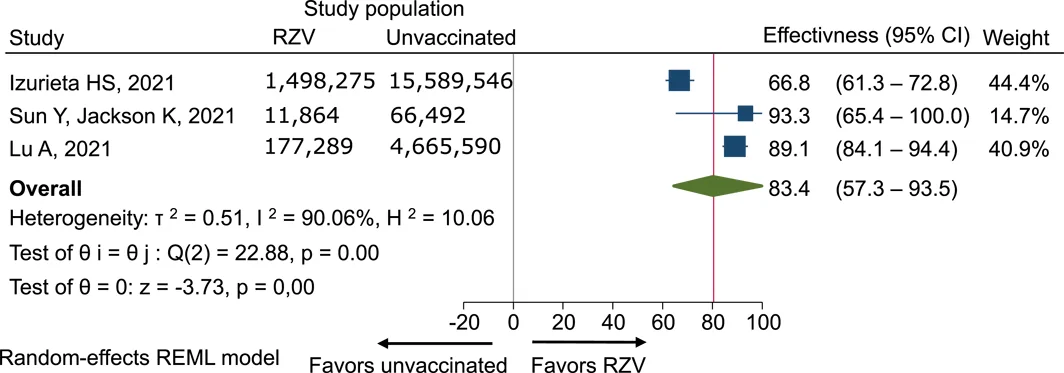
Meta‐analyses of VE against HZO in NRSIs. Estimates are adjusted vaccine‐effectiveness estimates reported by the included non‐randomized studies.

Post‐hoc analyses of the ZOE‐trials identified one HZO case in the vaccine group and seven in the control group over 3.9 years, corresponding to a VE against HZO of 86% with very wide confidence intervals (95% CI: −117 to 98) (moderate CoE) [43]. Meta‐analysis showed that RZV provided a substantial reduction in the risk of HZO in NRSIs [53, 54, 59] (Figure 6). The overall VE against HZO across age groups was 83.4% (95% CI: 57.3–93.5) (high CoE) but heterogeneity was substantial (*I* ^2^ = 90.0%). When excluding a study involving immunocompromised patients [53], the estimated vaccine effectiveness (VE) against HZO was 89.3% (95% CI: 83.5–93.1; *I* ^2^ 0.0%), (data not shown). One NRSI examined recurrence of HZO after RZV. However, due to few events, the results are uncertain [62].

### 3.7 Efficacy and Effectiveness Against Reduced Quality of Life, Pain, and Other Complications

Post‐hoc analyses of the ZOE‐trials showed 98.4% protection (95% CI: 92.2–100) against reduced quality of life among participants who developed HZ (Figure 5) [44]. RZV also appeared to reduce pain duration, though estimates were not statistically significant due to few HZ cases. In ZOE‐70, VE in reducing analgesic use was 39.6% (95% CI: 10.8–64.8) and 49.3% (95% CI: 2.9–73.5) in reducing treatment duration [45]. Protection against complications other than PHN was 93.7% (95% CI: 59.5–99.9) [43], and long‐term follow‐up showed 92.8% protection (95% CI: 71.6–99.2) against complications other than PHN [42].

### 3.8 Efficacy and Effectiveness Against Hospitalisation and Death

Across the ZOE‐trials, five hospitalizations were reported, all in the placebo group, and no deaths [43]. Three NRSIs assessed mortality after RZV [23, 52, 63]. Helm et al. 2023 [52] found a reduced mortality (adjusted RR 0.70; 95% CI: 0.57–0.88) in the first three years, whereas Taquet et al. 2024 [23], analysing the same register, found no reduction after four years (restricted mean time lost (RMTL) ratio 0.98; 0.95–1.01). Xu et al. 2025 [63], studying individuals with prior HZ, found no difference in mortality during the first year (adjusted HR 0.64; 95% CI: 0.32–1.25) (very low CoE, see Table S3).

### 3.9 Effectiveness Against Dementia

The four NRSIs [23, 51, 58, 61] investigating RZV and dementia all found an association between RZV and reduced risk of dementia. Three of these studies [51, 58, 61] relied on largely the same U.S. registry data source (Optum), from the same period. To avoid double‐counting participants and over‐weighting a single data source, these studies were not pooled together. Polisky et al. 2025 [58] was selected as the primary Optum‐based source because it was the most recent analysis, included extended follow‐up and used an active comparator. Tang et al. 2025 [61] was retained for descriptive comparison and sensitivity analysis.

**FIGURE 7:**
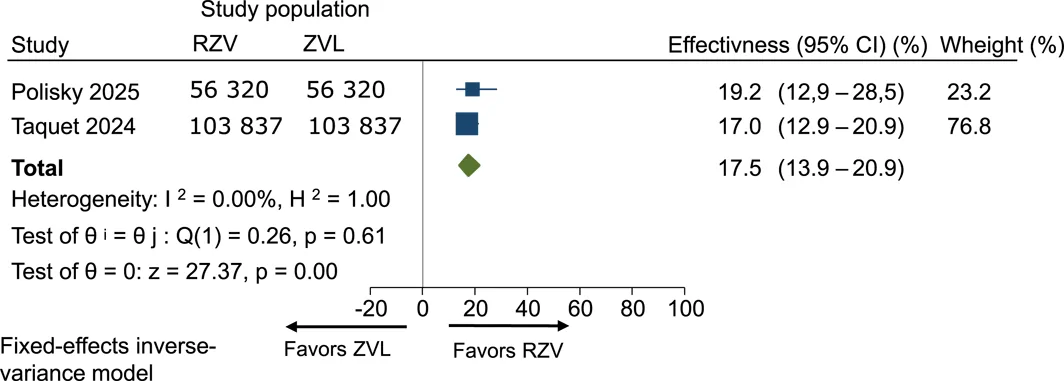
Direct comparison of RZV versus ZVL for dementia. The figure shows the relative association between RZV (recombinant zoster vaccine) and dementia‐related outcomes, compared with the older ZVL (live attenuated zoster vaccine).

Polisky et al. 2025 [58] found that receiving multiple doses (two or more) of RZV was associated with a significantly reduced risk of dementia compared with receiving a pneumococcal vaccine (PPSV23), with the largest reduction at 3 years (27.4%) and a smaller reduction at 5 years (16.9%) (Table S5a). Using stricter diagnostic criteria for dementia (both relevant ICD codes and prescription of dementia medication), the 5‐year results were not statistically significant [58]. Tang et al. 2025 [61] reported similar findings when comparing RZV recipients with unvaccinated individuals (adjusted VE of two RZV doses: 32% (95% CI 30–33, one dose VE: 11% (95% CI 8–13)) (Table S5b). When comparing RZV and ZVL, Polisky et al. 2025 [58] found a significantly lower dementia risk after RZV at 5 years (17.5%, 95% CI 12–31). Taquet et al. 2024 [23] found that RZV recipients had a 17% longer time to dementia diagnosis (164 additional days) than ZVL recipients. The pooled relative effect corresponded to a 17.6% reduction in dementia‐related outcomes after RZV compared with ZVL (95% CI 14.3–21.6; low CoE) (Figure 7). A sensitivity analysis replacing Polisky et al. 2025 [58] with Tang et al. 2025 [61] found a reduction in dementia of 14.0% (95% CI 11.4–16.6) (data not shown).

### 3.10 Effectiveness Against Cardiovascular Disease

Four NRSIs [52, 55, 56, 57, 63] and one post‐hoc analysis of the ZOE trials [46] assessed RZV protection against cardiovascular disease, comparing 677,700 vaccinated with 1,336,302 unvaccinated individuals. Due to differing follow‐up times, number of doses, and prior HZ history, we did not conduct meta‐analyses. Follow‐up periods varied widely: 53 months [46], 36 months [52], 23 months [63], 90 days [56, 57], and 42 days [55]. One study examined cardiovascular disease as a combined outcome [63]; the others assessed myocardial infarction and stroke separately, including one study with separate publications for each outcome [56, 57]. In a case control study among U.S. veterans, one dose of RZV was associated with an 18% lower myocardial infarction risk and a 43% lower stroke risk during the first month after HZ [56, 57]. In another population of individuals who developed HZ after age 50, prior RZV vaccination was associated with a 21% lower cardiovascular risk during the first year, the study did not adjust for higher smoking rates among the unvaccinated [63]. One study found that RZV reduced the risk of myocardial infarction by 27% compared with no vaccination, with no effect on stroke, and no difference compared with ZVL [52]. Two studies designed to assess the safety of RZV reported cardiovascular events, one using registry data [55] and the other using data from the ZOE studies [46]. No significant reduction in myocardial infarction was found in the ZOE post‐hoc study [46], and the NRSI reported no reduction in stroke but an 18% reduction in myocardial infarction [55] (Table S6). CoE was assessed as very low for the cardiovascular disease outcomes due to risk of bias in NRSIs and inconsistent findings.

## 4 Discussion

High certainty evidence indicates that RZV provides very high protection against HZ in immunocompetent adults aged ≥ 50 years (∼95% in RCTs; 81% in observational studies), with a possible trend towards slightly lower effectiveness with increasing age. Nearly all the included studies had a low risk of bias for this outcome (Table S2). For NRSIs assessing HZ, the between‐study heterogeneity was substantial (*I* ^2^ = 97.9%) and the prediction interval was wide, corresponding to a plausible VE range of 35.3%–94.3%. This heterogeneity may reflect differences in study populations, variation in follow‐up time, outcome ascertainment, handling of prior ZVL vaccination, and residual confounding. In particular, the lower estimates from Izurieta et al. 2021 [53] and Zerbo et al. 2024 [64] likely reflect older or broader clinical populations and inclusion of immunocompromised or corticosteroid‐exposed individuals. In a sensitivity analysis excluding these two studies the heterogeneity decreased to *I* ^2^ 0.0%.

Among vaccinated individuals who develop HZ, RZV is associated with reduced loss of quality of life and analgesic use. The vaccine also substantially reduces the risk of HZO, although the estimates are imprecise and heterogeneity in pooled HZO results is high (*I* ^2^ = 90.0%), the heterogeneity decreased to *I* ^2^ 0.0% excluding Zerbo et al. 2024 [64]. The vaccine likely reduces risk of PHN, but this is based on few studies with low event numbers for PHN. A post‐hoc analysis of the ZOE‐studies show consistent protection from RZV across sex, ethnicity and geographic regions [66]. Long‐term follow‐up suggests durable protection for at least 11 years, although CoE is lower due to the reliance of historical controls [42]. Emerging immunogenicity data indicate that both humoral and cellular immunity remain well above baseline for 10–20 years [67], but further studies are needed to clarify the potential need for booster doses. Despite the high vaccine efficacy, a relatively large number of individuals need to be vaccinated with RZV to prevent a single case of HZ. We found that, depending on age, the NNV is 33–36 to prevent one case of HZ over the initial 3–4 years, and NNV 11 to prevent one case of HZ over 11 years. The estimates for the initial 3–4 years are consistent with those reported in two other systematic reviews [37, 38], but higher than those observed in a third systematic review [34]. Higher NNVs are required to prevent one case of PHN or HZO (data not shown), which is consistent with findings from other systematic reviews for RZV [37, 38].

Protection after a single dose was not evaluated in the pivotal trials. However, evidence from NRSIs indicates that two doses of RZV provide stronger and more durable protection than a single dose, particularly among older adults and immunosuppressed individuals [53, 64, 68]. Zerbo et al. (2024) reported that one dose provided 70% effectiveness against HZ during the first year, with substantial decline thereafter, whereas protection after two doses remained stable for several years [64]. Izurieta et al. (2021) observed vaccine effectiveness of 57% against HZ after one dose, and 70% after two doses (median follow‐up 3 and 7 months respectively). They also evaluated dosing intervals up to 1 year and found that longer intervals did not reduce effectiveness [53]. This is consistent with a recent study by Tseng et al. (2025), which found VE against HZ of 60% after one dose and 74% after two doses, with a mean follow‐up of 2.5 years [68].

Jegede et al. (2025) reported findings suggesting that RZV may reduce the risk of recurrent HZ; however, these results may not be generalisable to individuals with multiple previous episodes or recent HZ [69]. As most studies excluded individuals with prior HZ, the evidence for this group remains limited. Further studies are needed to determine whether RZV should be offered to individuals with recurrent episodes, persistent chronic HZ symptoms, or recent HZ (within the past 6 months).

The included NRSIs show mixed results regarding the effectiveness of RZV against cardiovascular disease, with both inconsistent findings and a significant risk of bias (e.g., incomplete adjustment for smoking, blood pressure, BMI, and lipid levels). Overall, the current evidence does not support a clear reduction in cardiovascular risk. This is consistent with heterogeneous findings for ZVL [70], including a recent natural experiment study from Wales that found no cardiovascular benefit [71, 72].

Several observational studies have reported a reduced risk of dementia following vaccination with either ZVL or RZV [21, 22, 23, 51, 58, 61, 71, 72, 73, 74, 75, 76, 77, 78, 79, 80, 81, 82] (Figure S1). Evidence suggesting that RZV may reduce dementia risk comes exclusively from non‐randomized studies showing lower dementia incidence among vaccinated individuals compared with recipients of ZVL, other vaccines, or no vaccination. The GRADE certainty of this evidence is low, mainly because of serious concerns about a possible healthy vaccinee bias, residual confounding, and other limitations inherent to observational studies. The short interval between vaccination and dementia diagnosis, together with the rapid emergence of differences in incidence, raises concerns that the observed associations may partly reflect selection bias rather than a true biological effect [79]. Similar findings reported for other vaccines (e.g., RSV vaccine for older individuals) further support the possibility of residual confounding [76, 81, 83]. Natural experiments exploiting the age‐based rollout of ZVL‐vaccination in Wales, Australia, and Canada have shown consistent, modest reductions in dementia risk among those eligible for ZVL [71, 72, 77, 78]. In contrast, a recent preprint studying a UK population as a natural experiment did not find any reduction in hospital‐coded dementia diagnosis [84]. In the absence of randomized controlled trials, causality remains uncertain. Therefore, any potential reduction in dementia risk should be regarded as a possible secondary benefit, while the primary purpose of RZV remains prevention of HZ and PHN. Ongoing studies, including a pragmatic clinical trial in Denmark, a clinical trial in Finland and a planned randomized trial in Norway, may provide higher‐certainty evidence in the future [85, 86, 87, 88].

Our search was updated through 1 April 2025. Since then, two NRSIs on the effectiveness of RZV against HZ [68, 89] and one study on dementia [82] have been published. We consider it unlikely that these studies would materially alter the effect estimates for HZ or dementia. The two NRSIs report VE against HZ of approximately 74% and 85% following RZV [68, 89], consistent with our findings. All of the systematic reviews identified in our search were in consistent with our conclusions regarding VE against HZ, HZO and PHN [33, 34, 35, 36, 37, 38]. The results of this systematic review are based on RCTs and large NRSIs, which show consistent findings. Key outcomes were evaluated using the GRADE approach, with several outcomes rated as high CoE, supporting strong confidence in the results.

## 5 Conclusions

Two doses of RZV provide very strong protection against HZ in adults aged 50 years and older. The vaccine substantially reduces the burden of HZ‐related disease and markedly lowers the risk of HZO, and likely also PHN. Available data indicates durable protection for at least 11 years. At present, it remains unclear whether RZV influences the risk of dementia, cardiovascular disease or overall mortality. Any potential protection against dementia should be regarded as a possible secondary benefit to the primary goal of RZV; prevention of HZ and its associated complications.

The findings are relevant for health care professionals and policymakers when considering vaccine recommendations and reimbursement decisions. However, further studies are needed to clarify the potential protective effects of RZV against cardiovascular disease and dementia, the potential need for booster doses, and whether RZV should be offered to individuals with recurrent episodes, persistent chronic HZ symptoms, or recent HZ (within the past 6 months).

## Author Contributions

All the authors were involved in planning the work. E.H., K.J, I.H.T., S.L.S, H.N, A.E, J.Ø and L.K.J. contributed in with selection of included studies and assessment of the included studies. Analysis was done by J.Ø, L.K.J, I.H.T and E.H with the contribution by the other authors. All authors contributed to writing and completed the manuscript. All authors approved the submitted version.

## Funding

This work was funded by the Norwegian Institute of Public Health (NIPH) and the Public Health Agency of Sweden.

## Conflicts of Interest

The authors declare no conflicts of interest.

## Supporting information


**Figure S1:** Observational estimates of the association between herpes zoster vaccination and dementia‐related outcomes. Forest plot of reported relative reductions in dementia‐related outcomes after herpes zoster vaccination or herpes zoster exposure, stratified by vaccine type and comparator. Estimates are shown as reported or transformed to percentage reduction where applicable. The figure includes studies of recombinant zoster vaccine (RZV), zoster vaccine live (ZVL), combined or unspecified zoster vaccination. Outcomes include all‐cause dementia, Alzheimer’s disease, and vascular dementia. Because the included studies differ in design, comparator group, outcome definition, follow‐up time, and effect measure, estimates should be interpreted as descriptive and not directly comparable across all rows. Natural‐experiment absolute risk differences from Australia and Canada were converted to approximate relative risk reductions. AD, Alzheimer’s disease; CI, confidence interval; PPSV23, 23‐valent pneumococcal polysaccharide vaccine; RMTL, restricted mean time lost; RZV, recombinant zoster vaccine; Tdap, tetanus, diphtheria and acellular pertussis vaccine; VE, vaccine effectiveness; VHA, Veterans Health Administration; ZVL, live attenuated zoster vaccine.

**Table S1:** Characteristics of included studies.

**Table S2:** (a) Risk of biasin RCT (RoB 2). (b) Risk of bias in observational studies (ROBINS‐I).

**Table S3:** GRADE evidence tables.

**Table S4:** VE against HZ in different age groups from RCTs.

**Table S5:** (a) VE against dementia after propensity score matching (from Polisky et al. 2025 (58)). (b) VE against dementia (from Tang et al. 2025 (61)).

**Table S6:** Protective effects of RZV vs unvaccinated against cardiovascular diseases in adults above 50 or 65 years.

## Data Availability

The data that support the findings of this study are available from the corresponding author upon reasonable request.
