# Supplementary material for: Efficacy and Effectiveness of the Recombinant Zoster Vaccine Against Herpes Zoster, Herpes Zoster Ophthalmicus, Postherpetic Neuralgia and Dementia: A Systematic Review and Meta‐Analysis
Source: Rev Med Virol. 2026 Sep 16;36(5):e70203. doi: 10.1002/rmv.70203 (PMC13581344; doi:10.1002/rmv.70203)

Study	Exposure	Follow up	Outcome
Harris 2023 (51)	RZV (2 doses) vs Unvaccinated	2 y	Alzheimer’s disease
Harris 2023 (51)	RZV (any dose) vs Unvaccinated	2 y	Alzheimer’s disease
Polisky 2025 (58)	RZV (2+ doses) vs PPSV23	3 y	All dementia
Polisky 2025 (58)	RZV (2+ doses) vs PPSV23	5 y	All dementia
Rayens 2026 (82)	RZV (2+ doses) vs ZVL	5 y	All dementia
Rayens 2026 (82)	RZV (2 doses) vs Tdap	3 y	All dementia
Rayens 2026 (82)	RZV (2 doses) vs Unvaccinated	3 y	All dementia
Rayens 2026 (82)	RZV (female) vs Unvaccinated	3 y	All dementia
Schwab 2025 (65)	RZV (male) vs Unvaccinated	3 y	All dementia
Schwab 2025 (65)	RZV (2+ doses) vs PPSV23	3 y	All dementia
Schwab 2025 (65)	RZV (2+ doses) vs PPSV23	5 y	All dementia
Schwab 2025 (65)	RZV (2+ doses) vs ZVL	3 y	All dementia
Tang 2025 (61)	RZV (2+ doses) vs ZVL	5 y	All dementia
Tang 2025 (61)	RZV (1 dose) vs Unvaccinated	4 y	All dementia
Taquet 2024 (23)	RZV (2 doses) vs Unvaccinated	5 y	All dementia
Taquet 2024 (23)	RZV vs ZVL	6 y	All dementia
Taquet 2024 (23)	RZV vs ZVL (pre-COVID HR)		All dementia
Taquet 2024 (23)	RZV (female) vs ZVL	6 y	All dementia
Harris 2023 (51)	RZV (male) vs ZVL	6 y	All dementia
Pomirchy 2026 (77)	RZV + ZVL vs Unvaccinated	2 y	Alzheimer’s disease
Douros 2023 (79)	Any HZ vaccine vs Unvaccinated	8 y	Alzheimer’s disease
Eyting 2025 (76)	ZVL eligibility vs Not eligible	6 y	All dementia
Eyting 2025 (76)	Shingles (HZ) vs Unvaccinated	10 y	All dementia
Harris 2023 (51)	ZVL eligibility vs Not eligible	7 y	All dementia
Harris 2023 (51)	ZVL eligibility, ITT vs Not eligible	7 y	All dementia
Lophatananon 2023 (80)	ZVL alone vs Unvaccinated	8 y	Alzheimer’s disease
Polisky 2025 (58)	ZVL vs Unvaccinated	12 y	All dementia
Pomirchy 2025 (77)	ZVL vs PPSV23	3 y	All dementia
Scherrer2023 (74)	ZVL vs PPSV23	5 y	All dementia
Scherrer 2023 (74)	ZVL eligibility vs Not eligible	7 y	All dementia
Scherrer 2023 (74)	ZVL (MarketScan) vs Unvaccinated	3 y	All dementia
Scherrer 2023 (74)	ZVL (MarketScan) vs Unvaccinated	3 y	Alzheimer’s disease
Schnier 2022 (75)	ZVL (VHA) vs Unvaccinated	8 y	All dementia
Schnier 2022 (75)	ZVL (VHA) vs Unvaccinated	8 y	Alzheimer’s disease
Schnier 2022 (75)	ZVL vs Unvaccinated	7 y	All dementia
Schnier 2022 (75)	ZVL vs Unvaccinated	7 y	Alzheimer’s disease
Schnier 2022 (75)	ZVL vs Unvaccinated	7 y	Vascular dementia
Schwab 2025 (65)	ZVL, no shingles vs Unvaccinated	7 y	All dementia
Schwab 2025 (65)	ZVL, shingles vs Unvaccinated	7 y	All dementia
Wiemken 2023 (76)	ZVL vs PPSV23	3 y	All dementia
Wiemken 2023 (76)	ZVL vs PPSV23	5 y	All dementia
	ZVL (MarketScan) vs Unvaccinated	3 y	All dementia
	ZVL (VHA) vs Unvaccinated	7 y	All dementia

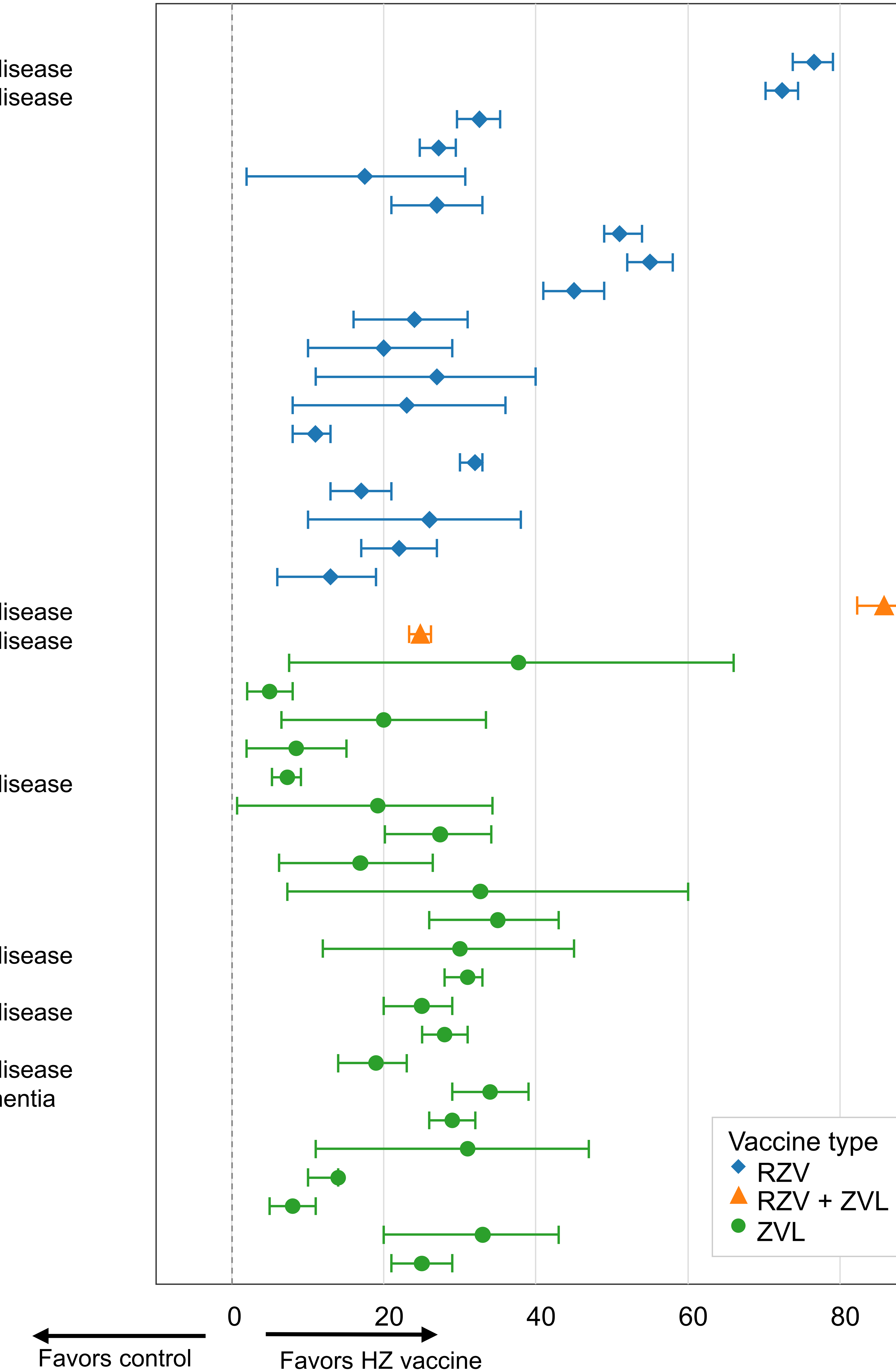

Supplement: Supplementary file 1 [file RMV-36-e70203-s003.pdf]
