# Supplementary material for: Efficacy and Effectiveness of the Recombinant Zoster Vaccine Against Herpes Zoster, Herpes Zoster Ophthalmicus, Postherpetic Neuralgia and Dementia: A Systematic Review and Meta‐Analysis
Source: Rev Med Virol. 2026 Sep 16;36(5):e70203. doi: 10.1002/rmv.70203 (PMC13581344; doi:10.1002/rmv.70203)
Supplement: Supplementary file 2 [file RMV-36-e70203-s001.docx]

**Supplementary table S5a:** VE against dementia after propensity score matching (from Polisky et al. 2025 (58))

| Comparing | Follow-up | Relative risk (95% CI) | N (per kohort, matched) |
| --- | --- | --- | --- |
| RZV (2+ doses) vs. PPV23 | 3 years | 0,73 (0,66–0,80) | 234 309 |
| RZV (2+ doses) vs. PPV23 | 5 years | 0,83 (0,74–0,94) | 234 309 |
| RZV (2+ doses) vs. RZV (1 dose) | 3 years | 0,76 (0,71–0,82) | 416 765 |
| RZV (2+ doses) vs. RZV (1 dose) | 5 years | 0,81 (0,74–0,88) | 416 765 |
| RZV (2+ doses) vs. ZVL | 3 years | 0,89 (0,74–1,08) | 56 320 |
| RZV (2+ doses) vs. ZVL | 5 years | 0,82 (0,69–0,98) | 56 320 |

**Supplementary table S5b:** VE against dementia (from Tang et al. 2025 (61))

| Comparing | Follow-up | Relative risk (95% CI) | N (RZV vs unvaccinated) |
| --- | --- | --- | --- |
| RZV (1+ doses) vs. unvaccinated | 48 months | 0,89 (0,87–0,92) | 3 835 9668 vs 206 297 |
| RZV (2+ doses) vs. unvaccinated | 60 months | 0,68 (0,67–0,70) | 3 835 9668 vs 460 413 |
| RZV (2+ doses) vs. ZVL |  | 0,88 (0,85–0,92) |  |
