# Supplementary material for: Efficacy and Effectiveness of the Recombinant Zoster Vaccine Against Herpes Zoster, Herpes Zoster Ophthalmicus, Postherpetic Neuralgia and Dementia: A Systematic Review and Meta‐Analysis
Source: Rev Med Virol. 2026 Sep 16;36(5):e70203. doi: 10.1002/rmv.70203 (PMC13581344; doi:10.1002/rmv.70203)
Supplement: Supplementary file 3 [file RMV-36-e70203-s002.docx]

**Supplementary table S4:** VE against HZ in different age groups from RCTs

| Age | Proano et al (41), 15,2 months follow-up  (95 % CI) | Lal et al (39), ZOE 50,  3.2 years follow-up  (95 % CI) | Cunningham et al (40), ZOE 70, 3,7 years follow-up  (95 % CI) | Cunningham et al (40), pooled ZOE 50/70, 3,8 years follow-up  (95 % CI) | Strezova et al (42), ZOE LTFU,  11 years follow-up  (95 % CI) |
| --- | --- | --- | --- | --- | --- |
| 50-59 years | 100  (89,3 - 100) | 96,6  (89,6 - 99,3) | - | - | 86,7  (75,6 – 93,4) |
| 60-69 years | 100  (89,3 - 100) | 97,4  (90,1 - 99,7) | - | - | 87,1  (74,2 - 94,4) |
| 70-79 years | - | - | 90,0  (83,5 - 94,4) | 91,3  (86,8 – 94,5) | 71,9  (60,1 – 80,6) |
| ≥ 70 years | 100  (60,3 - 100) | 97,9%  (87,9 - 100) | 89,8  (84,2 – 93,7) | 91,3  (86,0 – 94,9) | 73,2  (62,9 - 80,9) |
| ≥ 80 years | - | - | 89,1  (74,6 - 96,2) | 91,4  (80,2 - 97,9) | 75,9  (43,7 - 91,1) |

CI; confidence interval
