# Supplementary material for: Efficacy and Effectiveness of the Recombinant Zoster Vaccine Against Herpes Zoster, Herpes Zoster Ophthalmicus, Postherpetic Neuralgia and Dementia: A Systematic Review and Meta‐Analysis
Source: Rev Med Virol. 2026 Sep 16;36(5):e70203. doi: 10.1002/rmv.70203 (PMC13581344; doi:10.1002/rmv.70203)
Supplement: Supplementary file 4 [file RMV-36-e70203-s004.docx]

**Supplementary table S3:** GRADE evidence tables

We created a summary of findings table using the following outcomes: cumulative incidence of herpes zoster, herpes zoster complication and latest real-world data on long term protection of RZV against HZ and additional outcomes, such as cardiovascular disease, mortality and dementia. We used the five GRADE considerations (study limitations, consistency of effect, imprecision, indirectness, and publication bias), to assess the certainty of evidence as it relates to the studies that contribute data to the meta-analyses for the prespecified outcomes. We used the methods and recommendations described in Section 8.5 and Chapter 12 of the Cochrane Handbook for Systematic Reviews of Interventions, using GRADEpro GDT soſtware. We justified all decisions to downgrade or upgrade the certainty of studies using footnotes and made comments to aid the reader's understanding of the review where necessary.

Supplementary table S3a

|  | | | | | | |
| --- | --- | --- | --- | --- | --- | --- |
| **Recombinant Herpes Zoster vaccine (RZV) compared to no vaccine for reducing herpes zoster burden in elderly** | | | | | | |
| **Patient or population:** reducing herpes zoster burden in elderly  **Setting:** Community  **Intervention:** Recombinant Herpes Zoster vaccine (RZV)  **Comparison:** no vaccine | | | | | | |
| Outcomes | **Anticipated absolute effects^*^** (95% CI) | | Relative effect (95% CI) | № of participants (studies) | Certainty of the evidence (GRADE) | Comments |
|  | **Risk with no vaccine** | **Risk with Recombinant Herpes Zoster vaccine (RZV)** |  |  |  |  |
| Herpes zoster (HZ) follow-up: mean 3.2 years | 28 per 1 000 | **1 per 1 000** (4 to 0) | RR 0.049 (0.016 to 0.148) | 102 562 (3 RCTs)^1,2,3^ | ⨁⨁⨁⨁ High^a^ | VE 95.1 (95% CI 85.2 - 98.4) .  Not number of participants but person years (PY) |
| Herpes zoster (HZ) follow-up: median 11 years | 58 per 1 000 | **7 per 1 000** (9 to 6) | RR 0.123 (0.099 to 0.151) | 27 916 (1 RCT)^4^ | ⨁⨁◯◯ Low^b^ | VE 87.7 (95% CI 84.9-90.1). |
| Herpes zoster (HZ) follow-up: mean 22 months | 9 per 1 000 | **2 per 1 000** (3 to 2) | RR 0.199 (0.132 to 0.261) | 23 768 212 (5 non-randomised studies)^5,6,7,8,9^ | ⨁⨁⨁⨁ High^c^ | adjusted VE 80.1 % (95 CI 73.9 - 86.8).  Not number of participants but person years (PY). |
| Postherpatic neuralgia (PHN) follow-up: mean 3.8 years | 1 per 1 000 | **0 per 1 000** (0 to 0) | RR 0.09 (0.03 to 0.24) | 106 717 (1 RCT)^2^ | ⨁⨁⨁◯ Moderate^d^ | VE 91.0 (95% CI 76.0-97.0) |
| Postherpatic neuralgia (PHN) follow-up: mean 7 months | 1 per 1 000 | **0 per 1 000** (0 to 0) | RR 0.24 (0.18 to 0.31) | 20 116 890 (2 non-randomised studies)^5,6^ | ⨁⨁⨁◯ Moderate^e^ | VE 76 % (95% CI 68.4 – 81.8)^6^.  Not number of participants but person years (PY). |
| Herpes zoster ophthalmicus (HZO) follow-up: mean 3.8 years | 0 per 1 000 | **0 per 1 000** (0 to 0) | RR 0.14 (0.02 to 1.17) | 106 755 (1 RCT)^2^ | ⨁⨁⨁◯ Moderate^d^ | VE 86.0 (95% CI -117.0 - 98.0). |
| Herpes zoster ophthalmicus (HZO) follow-up: mean 1.3 years | 1 per 1 000 | **0 per 1 000** (0 to 0) | RR 0.17 (0.06 to 0.43) | 22 009 056 (3 non-randomised studies)^6,7,10^ | ⨁⨁⨁⨁ High | adjusted VE 83.4% (95% CI 57.3 - 93.5).  Not number of participants but person years (PY). |
| Mortality follow-up: range 1 years to 4 years | Two studies from same population (registries) differs in results, on show a reduction after three years and on study show no association. The third study show no association after 1 year. | |  | (3 non-randomised studies)^11,12,13^ | ⨁◯◯◯ Very low^f,g^ | The evidence from three observational studies are conflicting and of low certainty, and more studies are needed to conclude on this outcome |
| Myocardial infarction (MI) follow-up: range 30 days to 4 years | One post-hoc (ZOE study) show no difference. From the four observational studies, three shows a small reduced risk after 30 days, or 2 or 3 years. One study show a reduction in all cardiovascular disease after one year. Se table 11. | |  | (5 non-randomised studies)^12,14,15,16,17^ | ⨁◯◯◯ Very low^f,h^ | The evidence is from four observational studies show a small reduction, while a post-hoc study show no reduction, the evidence is of low certainty, and more studies are needed to conclude on this outcome |
| Stroke follow-up: range 30 days to 3 years | Three studies show no difference between the groups after 3 or 4 years, and one study show an small reduction after 30 days. One study show a reduction in all cardiovascular disease after one year. Se table 11. | |  | (4 non-randomised studies)^11,12,15,16^ | ⨁◯◯◯ Very low^f,i^ | The evidence from from observational studies are conflicting and of low certainty, and more studies are needed to conclude on this outcome |
| ***The risk in the intervention group** (and its 95% confidence interval) is based on the assumed risk in the comparison group and the **relative effect** of the intervention (and its 95% CI). **CI:** confidence interval; **RR:** risk ratio | | | | | | |
| **GRADE Working Group grades of evidence** **High certainty:** we are very confident that the true effect lies close to that of the estimate of the effect. **Moderate certainty:** we are moderately confident in the effect estimate: the true effect is likely to be close to the estimate of the effect, but there is a possibility that it is substantially different. **Low certainty:** our confidence in the effect estimate is limited: the true effect may be substantially different from the estimate of the effect. **Very low certainty:** we have very little confidence in the effect estimate: the true effect is likely to be substantially different from the estimate of effect. | | | | | | |

#### Explanations

a. Different follow up time in the studies

b. Longterm follow up study of the ZOE studies, but the control arm receive vaccine after five years, therefore a historical control data arm was calculated by prevalence data. In addition: missing outcome-data.

c. Two studies had moderate risk of bias due to missing data, one of them (Florea et al) had additional risk related to substantial exclusion of participants that didn’t receive 2^nd^ dose as intended (deviation from intended intervention), the other (Zerbo et al) had potential for unmeasured immunosuppression status (confounding).

d. Few events

e. One study (Florea et al) moderate risk of bias due to missing data and substantial exclusion of participants that didn’t receive 2^nd^ dose as intended (deviation from intended intervention)

f. Risk of bias mainly due to confounding (i.e. no adjustment on smoking in most studies), missing data and selective reporting.

g.Two studies with the same population from the same register show different results. The third study shows no effect on mortality from RZV

h. One post hoc study shows no difference between the groups and three observational studies indicate a reduction in MI

i. Two studies show a reduction and two studies do not show a reduction

#### References

1.Lal, .2015 (1). 2.Cunningham, .2016 (2). 3.Proano, 2024 (3). 4. Strezova, 2025 (4). 5.Florea, 2024 (5). 6.Izurieta, .2021 (6). 7.Sun Y, Jackson.2021 (7). 8.Sun Y, Kim. 2021 (8)..9. Zerbo, .2024 (9). 10.Lu, 2021 (10). 11.Xu, 2025 (11). 12.Helm, 2023 (12). 13.Taquet, 2024 (13). 14. Nelson, 2023 (14).15. Parameswaran, 2023 (15, 16). 16.Lopez-Fauqued, 2019 (17).

| **Supplementary table S3b** | | | | | | |
| --- | --- | --- | --- | --- | --- | --- |
| **Recombinant Herpes Zoster vaccine (RZV) compared to Pnemoccocus vaccine (PPSV23) for reducing dementia in elderly** | | | | | | |
| **Patient or population:** reducing dementia in elderly  **Setting:** Community  **Intervention:** Recombinant Herpes Zoster vaccine (RZV)  **Comparison:** Pnemoccocus vaccine (PPSV23) | | | | | | |
| Outcomes | **Anticipated absolute effects^*^** (95% CI) | | Relative effect (95% CI) | № of participants (studies) | Certainty of the evidence (GRADE) | Comments |
|  | **Risk with Pnemoccocus vaccine (PPSV23)** | **Risk with Recombinant Herpes Zoster vaccine (RZV)** |  |  |  |  |
| Dementia assessed with: ICD10/9 follow-up: mean 3 years | 5 per 1 000 | **3 per 1 000** (4 to 3) | RR 0.73 (0.66 to 0.80) | 468 618 (1 non-randomised study)^1^ | ⨁⨁◯◯ Low^a,b^ | VE 27.4 % (95% CI 20.1-34.1) |
| Dementia  assessed with: ICD10 follow-up: mean 5 years | 6 per 1 000 | **5 per 1 000** (5 to 4) | RR 0.83 (0.74 to 0.94) | 468 618 (1 non-randomised study)^1^ | ⨁⨁◯◯ Low^a,b^ | VE 16,9 % (95% CI 6.2-26.4) |
| ***The risk in the intervention group** (and its 95% confidence interval) is based on the assumed risk in the comparison group and the **relative effect** of the intervention (and its 95% CI).  **CI:** confidence interval; **RR:** risk ratio | | | | | | |
| **GRADE Working Group grades of evidence** **High certainty:** we are very confident that the true effect lies close to that of the estimate of the effect. **Moderate certainty:** we are moderately confident in the effect estimate: the true effect is likely to be close to the estimate of the effect, but there is a possibility that it is substantially different. **Low certainty:** our confidence in the effect estimate is limited: the true effect may be substantially different from the estimate of the effect. **Very low certainty:** we have very little confidence in the effect estimate: the true effect is likely to be substantially different from the estimate of effect. | | | | | | |

#### Explanations

a. Moderate risk of bias according to ROBINs-I. Risk of bias due to missing data in lost to follow up and censoring.

b. Outcome indirectness due to the timing of their assessment. Short period of vaccine until the dementia diagnosis (10 days), difficult to address if the nonacute nature of dementia diagnosis early is related to vaccines is early detection bias (Polinsky 2025).

#### References

1.Polisky, 2025.

**Supplementary table S3c.**

|  | | | | | | |
| --- | --- | --- | --- | --- | --- | --- |
| **Recombinant Herpes Zoster vaccine (RZV) compared to Live herpes zoster vaccine (ZVL) for reducing dementia in elderly** | | | | | | |
| **Patient or population:** reducing dementia in elderly  **Setting:** Community  **Intervention:** Recombinant Herpes Zoster vaccine (RZV)  **Comparison:** Live herpes zoster vaccine (ZVL) | | | | | | |
| Outcomes | **Anticipated absolute effects^*^** (95% CI) | | Relative effect (95% CI) | № of participants (studies) | Certainty of the evidence (GRADE) | Comments |
|  | **Risk with Live herpes zoster vaccine (ZVL)** | **Risk with Recombinant Herpes Zoster vaccine (RZV)** |  |  |  |  |
| Dementia assessed with: ICD10 follow-up: mean 5 years | 8 per 1 000 | **6 per 1 000** (7 to 6) | RR 0.82 (0.79 to 0.86) | 320 314 (2 non-randomised studies)^1,2^ | ⨁⨁◯◯ Low^a,b^ | VE 17.5 % (95% CI 13.9-20.9) |
| ***The risk in the intervention group** (and its 95% confidence interval) is based on the assumed risk in the comparison group and the **relative effect** of the intervention (and its 95% CI).  **CI:** confidence interval; **RR:** risk ratio | | | | | | |
| **GRADE Working Group grades of evidence** **High certainty:** we are very confident that the true effect lies close to that of the estimate of the effect. **Moderate certainty:** we are moderately confident in the effect estimate: the true effect is likely to be close to the estimate of the effect, but there is a possibility that it is substantially different. **Low certainty:** our confidence in the effect estimate is limited: the true effect may be substantially different from the estimate of the effect. **Very low certainty:** we have very little confidence in the effect estimate: the true effect is likely to be substantially different from the estimate of effect. | | | | | | |

#### Explanations

a. Moderate risk of bias according to ROBINs-I. Risk of bias due to missing data in lost to follow up and censoring.

b. Outcome indirectness due to the timing of their assessment for one of the studies. Short period of vaccine until the dementia diagnosis (10 days), difficult to address if the nonacute nature of dementia diagnosis early is related to vaccines is early detection bias in one of the studies (Polisky 2025).

#### References

1.Polisky,2025. 2.Taquet,.2025.

References

1.	Lal H, Cunningham AL, Godeaux O, Chlibek R, Diez-Domingo J, Hwang SJ, et al. Efficacy of an adjuvanted herpes zoster subunit vaccine in older adults. N Engl J Med. 2015;372(22):2087-96.

2.	Cunningham AL, Lal H, Kovac M, Chlibek R, Hwang SJ, Diez-Domingo J, et al. Efficacy of the Herpes Zoster Subunit Vaccine in Adults 70 Years of Age or Older. N Engl J Med. 2016;375(11):1019-32.

3.	Alexandra Echeverria Proano D, Zhu F, Sun X, Zoco J, Soni J, Parmar N, et al. Efficacy, reactogenicity, and safety of the adjuvanted recombinant zoster vaccine for the prevention of herpes zoster in Chinese adults ≥ 50 years: A randomized, placebo-controlled trial. Hum Vaccin Immunother. 2024;20(1):2351584.

4.	Strezova A, Diez Domingo J, Cunningham AL, Eto T, Andrews C, Arns C, et al. Final analysis of the ZOE-LTFU trial to 11 years post-vaccination: efficacy of the adjuvanted recombinant zoster vaccine against herpes zoster and related complications. EClinicalMedicine. 2025;83:103241.

5.	Florea A, Sy L, Qian L, Ackerson B, Luo Y, Wu J, et al. Real-world effectiveness of recombinant zoster vaccine in self-identified Chinese individuals aged >=50 years in the United States. Human vaccines & Immunotherapeutics. 2024;20(1):2327145.

6.	Izurieta HS, Wu X, Forshee R, Lu Y, Sung HM, Agger PE, et al. Recombinant Zoster Vaccine (Shingrix): Real-World Effectiveness in the First 2 Years Post-Licensure. Clin Infect Dis. 2021;73(6):941-8.

7.	Sun Y, Jackson K, Dalmon CA, Shapiro BL, Nie S, Wong C, et al. Effectiveness of the recombinant zoster vaccine among Kaiser Permanente Hawaii enrollees aged 50 and older: A retrospective cohort study. Vaccine. 2021;39(29):3974-82.

8.	Sun Y, Kim E, Kong CL, Arnold BF, Porco TC, Acharya NR. Effectiveness of the Recombinant Zoster Vaccine in Adults Aged 50 and Older in the United States: A Claims-Based Cohort Study. Clin Infect Dis. 2021;73(6):949-56.

9.	Zerbo O, Bartlett J, Fireman B, Lewis N, Goddard K, Dooling K, et al. Effectiveness of Recombinant Zoster Vaccine Against Herpes Zoster in a Real-World Setting. Ann Intern Med. 2024;177(2):189-95.

10.	Lu A, Sun Y, Porco TC, Arnold BF, Acharya NR. Effectiveness of the Recombinant Zoster Vaccine for Herpes Zoster Ophthalmicus in the United States. Ophthalmology. 2021;128(12):1699-707.

11.	Wu PY, Wu HD, Chou TC, Sung FC. Varicella vaccination alters the chronological trends of herpes zoster and varicella. PLoS ONE. 2013;8(10):e77709.

12.	Helm MF, Khoury PA, Pakchanian H, Raiker R, Maczuga S, Foulke GT. Recombinant Zoster Vaccine Reduces 3-Year Cardiovascular Risk: Insights From a Multi-Centered Database. J Drugs Dermatol. 2023;22(12):1178-82.

13.	Taquet M, Dercon Q, Todd JA, Harrison PJ. The recombinant shingles vaccine is associated with lower risk of dementia. Nat Med. 2024;30(10):2777-81.

14.	Nelson JC, Ulloa-Perez E, Yu O, Cook AJ, Jackson ML, Belongia EA, et al. Active Postlicensure Safety Surveillance for Recombinant Zoster Vaccine Using Electronic Health Record Data. Am J Epidemiol. 2023;192(2):205-16.

15.	Parameswaran GI, Drye AF, Wattengel BA, Carter MT, Doyle KM, Mergenhagen KA. Increased Myocardial Infarction Risk Following Herpes Zoster Infection. Open Forum Infect Dis. 2023;10(4):ofad137.

16.	Parameswaran GI, Wattengel BA, Chua HC, Swiderek J, Fuchs T, Carter MT, et al. Increased Stroke Risk Following Herpes Zoster Infection and Protection With Zoster Vaccine. Clin Infect Dis. 2023;76(3):e1335-e40.

17.	Lopez-Fauqued M, Campora L, Delannois F, El Idrissi M, Oostvogels L, De Looze FJ, et al. Safety profile of the adjuvanted recombinant zoster vaccine: Pooled analysis of two large randomised phase 3 trials. Vaccine. 2019;37(18):2482-93.
