# Supplementary material for: Efficacy and Effectiveness of the Recombinant Zoster Vaccine Against Herpes Zoster, Herpes Zoster Ophthalmicus, Postherpetic Neuralgia and Dementia: A Systematic Review and Meta‐Analysis
Source: Rev Med Virol. 2026 Sep 16;36(5):e70203. doi: 10.1002/rmv.70203 (PMC13581344; doi:10.1002/rmv.70203)
Supplement: Supplementary file 5 [file RMV-36-e70203-s005.docx]

## Supplementary

**Supplemetary table S1:** Characteristics of included studies

| Publication | Study design and funding | Inclusion and exclusion criteria | Number of patients | Mean age ±SD | Intervention vs comparator | Selected outcomes relevant to SR protocol | Adjustments | Description of main results regarding effect |
| --- | --- | --- | --- | --- | --- | --- | --- | --- |
| RANDOMIZED CONTROLLED TRIALS RZV (VE = Vaccine efficacy) | | | | | | | | |
| Cunningham AL, et al. Efficacy of the Herpes Zoster Subunit Vaccine in Adults 70 Years of Age or Older. N Engl J Med. 2016 Sep 15;375(11):1019-32.  Multicountry | Phase 3 RCT at 163 sites in 18 countries/regions  1:1 randomized  Placebo-controlled  Triple blind  02.08.10 - 24.07.15  Funded by industry (GSK)  **FU**: 3,7 years | **Inclusion criteria TVC:**  Age ≥ 70 years  **Exclusion criteria TVC:**  Immunosuppressive condition  History of HZ  HZ or varicella vaccine  Life expectancy < 4 years  **Additional exclusion criteria for the MVC:**  HZ diagnosis ≤ 30 days after dose 2, only 1 dose of RZV. | **Total:**  ZOE-70 TVC:  13 900 (53 025* PY)  ZOE-70 MVC:  13 163 (48 573* PY)  **Vaccinated:**  ZOE-70 TVC:  6950 (26 753 PY)  ZOE-70 MVC:  6 541 (24 405 PY)  **Placebo:**  ZOE-70 TVC:  6950 (26 272 PY)  ZOE-70 MVC:  6 622 (24 168 PY) | 75,6 ±4,7 years (62-96) | 2 doses RZV I.M vs placebo | **Primary outcome:**  Efficacy against HZ ≥ 70 years  **Secondary outcome:**  Efficacy in the prevention of overall PHN  Efficacy in reducing the total duration of severe ‘worst’ HZ-associated pain  Efficacy in the reduction of overall and HZ-related mortality and hospitalizations  Efficacy in the reduction in incidence of HZ-associated complications  Efficacy in the reduction in use of pain medications  Safety  **Exploratory outcomes:**  Efficacy in reducing the severity of acute HZ-associated pain  Efficacy in improving quality of life  Efficacy in the mitigation of burden of illness caused by HZ  Evaluate vaccine induced humoral immune responses and anti-VZV neutralizing antibody titers | Not applicable | VE (95% CI) against **HZ by age-group** in years in ZOE-70 MVC (table 1):  Overall: 89,8 (84,2-93,7)  70-79: 90,0 (83,5-94,4)  ≥ 80: 89,1 (74,6-96,2)  VE% (95% CI) against **HZ by years since vaccination** in ZOE-70 MVC (table 1):  1 year: 97,0 (88,8-99,7)  2 years: 91,3 (79,9-96,9)  3 years: 81,6 (61,9-92,1)  4 years: 85,1 (64,4-94,9)  VE against **HZ** in ZOE-70 TVC available in table S2. |
|  | Same as above  **FU:** 3,8 years | **Pooled vaccinated cohort ≥ 50 years (“pooled ZOE-50/70”):** Participants in ZOE-50 ≥ 50 years in addition to ZOE-70 participants (used in analysis of PHN)  **Pooled vaccinated cohort ≥ 70 years (“pooled ZOE-70 analysis”):** Participants in ZOE-50 ≥ 70 years in addition to ZOE-70 participants.  Same inclusion- and exclusion criteria as above. | **Total:**  Pooled ZOE-50/70 TVC:  29 305 (115 951* PY)  Pooled ZOE-50/70 MVC:  27 916* (106 717* PY)  Pooled ZOE-70 TVC:  17 531* (66 710* PY)  Pooled ZOE-70 MVC:  16 596* (61 141* PY)  **Vaccinated:**  Pooled ZOE-50/70 TVC:  14 645 (57 915 PY)  Pooled ZOE-50/70 MVC:  13 881 (53 172 PY)  Pooled ZOE-70 TVC:  8 758 (HZ 33 638 PY/PHN 33 679 PY)  Pooled ZOE-70 MVC:  8 250 (30 726 PY)  **Placebo:**  Pooled ZOE-50/70 TVC:  14 660 (58 036 PY)  Pooled ZOE-50/70 MVC:  14 035 (53 545 PY)  Pooled ZOE-70 TVC:  8 773 (HZ 33 072 PY/PHN 33 684 PY)  Pooled ZOE-70 MVC:  8 346 (30 415 PY) | 75,5 ±4,7 years | 2 doses RZV I.M vs placebo | **Primary outcome:**  Efficcy against HZ and PHN ≥ 70 years  **Secondary outcome:**  Efficacy in the prevention of overall PHN  Efficacy in reducing the total duration of severe ‘worst’ HZ-associated pain  Safety  **Exploratory outcomes:**  Efficacy in reducing the severity of acute HZ-associated pain  Efficacy in improving quality of life  Efficacy in the mitigation of burden of illness caused by HZ  Evaluate vaccine induced humoral immune responses and anti-VZV neutralizing antibody titers | Not applicable | VE (95% CI) against **PHN by age-group** in years in pooled ZOE-50/70 MVC (table 1):  ≥ 70: 88,8 (68,7-97,1)  ≥ 50: 91,2 (75,9-97,7)  VE against **HZ** in Pooled ZOE-70 MVC, and VE against **PHN** **by age groups** in pooled ZOE-50/70 MVC available in table 1.  VE against **HZ** in Pooled ZOE-70 TVC, and VE against **PHN** in pooled ZOE-50/70 TVC available in table S2. |
| Curran D, et al. Quality of Life Impact of an Adjuvanted Recombinant Zoster Vaccine in Adults Aged 50 Years and Older. J Gerontol A Biol Sci Med Sci. 2019 Jul 12;74(8):1231-1238  Multicountry | ZOE-50 and  pooled ZOE-70  Assessments integrated in ZOE-50 and ZOE-70.  Funded by industry (GSK).  **FU ZOE-50:**  3,9 ± 0,7 years  **FU ZOE-70:**  3,7 ± 0,8 years | **Inclusion criteria ZOE-50:**  Age ≥ 50 years with ZBPI-score  **Inclusion criteria pooled vaccinated cohort ≥ 70 years (“pooled ZOE-70”) TVC:** Participants in ZOE-50 ≥ 70 years in addition to ZOE-70 participants, all with ZBPI-score  **Exclusion criteria TVC:**  See ZOE-50 and ZOE-70  **Additional exclusion criteria for the MVC:**  HZ diagnosis ≤ 30 days after dose 2, only 1 dose of RZV. | **Total:**  ZOE-50 MVC:  14 751  Pooled ZOE-70 MVC:  16 593  **Vaccinated:**  ZOE-50 MVC:  7 340  Pooled ZOE-70 MVC:  8 250  **Placebo:**  ZOE-50 MVC:  7 411  Pooled ZOE-70 MVC:  8 343 | **Vaccinated:**  ZOE-50:  61,9 (50-75)  Pooled ZOE-70:  76,2 (70-86)  **Placebo:**  ZOE-50:  62,9 (50-87)  Pooled ZOE-70:  75,8 (70-92) | 2 doses RZV I.M vs placebo | **Primary outcome:**  Efficacy against HZ burden of illness  (Least, worst and average pain)  (HZ burden of interference)  (Quality of life) | Not applicable | VE (95% CI) against **HZ burden of illness** in ZOE-50 MVC:  Overall: 98,4 (92,2-100)  50-59: 98,3 (83,8-100)  60-69: 98,1 (79,2-100)  ≥ 70: 98,9 (72,1-100)  VE (95% CI) against **HZ burden of illness** in pooled ZOE-70 MVC:  Overall: 92,1 (90,4-93,8)  70-79: 98,1 (92,5-97,7)  ≥ 80: 98,9 (77,2-87,2) |
| Curran D, et al. Recombinant Zoster Vaccine Is Efficacious and Safe in Frail Individuals. J Am Geriatr Soc. 2021 Mar;69(3):744-752.  Multicountry | Pooled ZOE-50/70  Secondary retrospective analysis to assess the baseline frailty status of participants in the ZOE-50 and ZOE-70 studies, at sites willing to participate.  Funded by industry (GSK).  **FU:** Approximately 4 years during the ZOE-studies | **Inclusion criteria Pooled vaccinated cohort ≥ 50 years (“pooled ZOE-50/70”) TVC:** Participants in ZOE-50 ≥ 50 years in addition to ZOE-70 participants  **Exclusion criteria TVC:**  See ZOE-50 and ZOE-70  **Additional exclusion criteria for the MVC:**  HZ diagnosis ≤ 30 days after dose 2, only 1 dose of RZV. | **Total:**  TVC: 26 976  (92% of the 29 305 persons in the pooled ZOE-50/70 TVC)  MCV: 25 715 (97 539* PY)  **Vaccinated**:  TVC: 13 487  MVC: 12 785 (48 989* PY)  **Placebo**:  TVC: 13 489  MVC: 12 930 (48 550* PY) | **Total**:  68,8 (48-96) years  **Vaccinated:** 68,8 (50-96) years  **Placebo:** 68,8 (48-95) years | 2 doses RZV I.M vs placebo | **Primary outcome:**  Frailty status  **Secondary outcomes – by frailty status:**  Efficacy against HZ  Efficacy against HZ burden of illness  (Immunogenicity)  (Reactogenicity)  Safety | Not applicable | VE (95% CI) against **HZ by frailty-group** in pooled ZOE-50/70 MVC (supplementary table S3):  Non-frail: 95,8 (91,6-98,2)  Pre-frail: 90,4 (84,4-94,4)  Frail: 90,2 (75,4-97,0)  Unknown: 100 (12,6-100,0)  VE against burden of illness by frailty-group available in supplementary table S3. |
| Dagnew AF, et al. The Adjuvanted Recombinant Zoster Vaccine in Adults Aged ≥65 Years Previously Vaccinated With a Live-Attenuated Herpes Zoster Vaccine. J Infect Dis. 2021 Oct 13;224(7):1139-1146.  Multicountry | Pooled ZOE-50/70  Post-hoc analysis of RZV's efficacy against HZ and safety profile in ZOE-50/70 participants who reported pre-existing potential immune-mediated diseases (pIMDs) at enrolment and were not on immunosuppressive therapies, at sites willing to participate.  Funded by industry (GSK)  **FU:** 4,4 years in the TVC | **Inclusion criteria: Pooled vaccinated cohort ≥ 50 years (“pooled ZOE-50/70”):** Participants in ZOE-50 ≥ 50 years in addition to ZOE-70 participants  with ≥ 1 potential immune-mediated disease (pIMD) at enrolment,  **Exclusion criteria TVC:**  See ZOE-50 and ZOE-70, no pIMD at enrolment  **Additional exclusion criteria for the MVC:**  HZ diagnosis ≤ 30 days after dose 2, only 1 dose of RZV. | **Total:**  TVC: 1 943  (6,6% of the 29 305 persons in the pooled ZOE-50/70 TVC)  MVC: 1 859 (7 021* PY)  **Vaccinated**:  TVC: 983  MVC: 936 (3 612 PY)  **Placebo:**  TVC: 960  MVC: 923 (3 409 PY) | **Vaccinated:** 68,8±9,6 years  **Placebo:** 69,4±9,5 years | 2 doses RZV I.M vs placebo | Efficacy against HZ in subset with pre-existing pIMDS at enrolment (MVC)  Safety in subset with pIMDs (TVC) | Not applicable | Overall VE (95% CI) against **HZ in subset with pIMDs** in pooled ZOE-50/70 MVC (table 2):  90,5(73,5-97,5)  VE (95% CI) against **HZ in subset with pIMDs by age-group** in pooled ZOE 50/70 MVC, years (table 2):  50-59: 92,8 (50,5-99,8)  60-69: 100,0 (54,9-100,0)  70-79: 84,4 (30,8-98,3)  ≥ 80: 86,2 (-13,5-99,7) |
| Kim JH, et al. Adjuvanted recombinant zoster vaccine decreases herpes zoster-associated pain and the use of pain medication across 3 randomized, placebo-controlled trials. Pain. 2023 Apr 1;164(4):741-748.  Multicountry    “ZOE –50/70/HSCT pain” | Post-hoc analysis of ZOE-50/70 and ZOE-HSCT  Funded by industry (GSK).  **FU:**  ZOE-50:  3,2 years    ZOE-70: 3,7 years    ZOE-HSCT (Autologous hematopoietic stem cell transplantation) | Patients with HZ-diagnosis in mTVC ZOE-50/70 and ZOE-HSCT    mTVC: modifies total vaccinated cohort | Total:    Vaccinated:  ZOE-50: 9  ZOE-70: 23  ZOE-HSCT: 49    Unvaccinated:  ZOE-50: 254  ZOE-70: 223  ZOE-HSCT: 135 | ZOE-50: 62,3 ± 9,0 years    ZOE-70:  75,6 ±4,7 years (62-96)    ZOE-HSCT | 2 doses RZV I.M vs placebo | Efficacy in reducing duration of clinically significant HZ-associated pain    Efficacy against use, and duration of use, of HZ-associated pain medication | Not applicable | VE (95% CI) against **duration of clinically significant HZ associated pain** in mTVC (table 1):  ZOE-50: 26,9 (-59,6-66,5)  ZOE-70: 28,4 (-17,7-56,4)  ZOE-HSCT: 38,5 (11,1-57,5)  Pooled **mean duration of HZ in days** difference between placebo and RZV: SMD 15.2 (P=0.1992)    VE (95% CI) against **use of HZ- associated pain medication** in mTVC (table 3):  ZOE-50: 11,7 (-19,4-53,6)  ZOE-70: 39,6 (10,8-64,8)  ZOE-HSCT: 6,2 (-18,8-27,8)    VE (95% CI) against **duration of use of HZ- associated pain medication** in mTVC (table 4):  ZOE-50: 29,4 (-73,7-67,4)  ZOE-70: 49,36 (2,9-73,5)  ZOE-HSCT:22,5 (-15,9-48,1) |
| Kovac M, et al. Complications of herpes zoster in immunocompetent older adults: Incidence in vaccine and placebo groups in two large phase 3 trials. Vaccine. 2018 Mar 14;36(12):1537-1541.  Multicountry | Pooled ZOE-50/70  Secondary analysis to explore RZV’s efficacy in reducing HZ-associated complications, mortality and hospitalizations in ZOE-50/70 participants.  Funded by industry (GSK)  **FU:**  ZOE-50:  3,9 ± 0,7 years  ZOE-70:  3,7 ± 0,8 years | **Inclusion criteria Pooled vaccinated cohort ≥ 50 years (“pooled ZOE-50/70”):** Participants in ZOE-50 ≥ 50 years in addition to ZOE-70 participants  **Inclusion criteria Pooled vaccinated cohort ≥ 70 years (“pooled ZOE-70 analysis”):** Participants in ZOE-50 ≥ 70 years in addition to ZOE-70 participants.  **Exclusion criteria TVC:**  See ZOE-50 and ZOE-70  **Additional exclusion criteria for the MVC:**  HZ diagnosis ≤ 30 days after dose 2, only 1 dose of RZV. | **Total:**  Pooled ZOE-50/70 MVC: 27 916* (106 692* PY)  Pooled ZOE-70 MVC:  16 596* (61 685* PY)  **Vaccinated**:  Pooled ZOE-50/70 MVC:  13 881 (53 173 PY)  Pooled ZOE-70 MVC:  8 250 (30 762 PY)  **Placebo**:  Pooled ZOE-50/70 MVC:  14 035 (53 582 PY)  Pooled ZOE-70 MVC:  8 346 (30 969 PY)  No data on TVC. | Not specified | 2 doses RZV I.M vs placebo | **Primary outcome:**  See ZOE-50/70  **Secondary outcomes:**  Efficacy against HZ-complications (not PHN)  Efficacy against HZ-related mortality  Efficacy against HZ-related hospitalisation | Not applicable | Overall VE (95% CI) against **HZ-related complications other than PHN** in pooled ZOE-50/70 MVC and pooled ZOE-70 MVC (table 3):  ≥ 50 years: 93,7(59,5-99,9)  ≥ 70 years: 91,6 (43,3-99,8)  VE (95% CI) against **HZ-related complications other than PHN by age group**, in pooled ZOE-50/70 MVC, years (table 3):  50-59: 100,0 (-3890,4-100,0)  60-69: 100,0 (-142,8-100,0)  70-79: 87,4 (5,9-99,8)  ≥ 80: 100,0 (-51,1-100,0)  VE against **HZ-related complications including PHN** in pooled ZOE-50/70 MVC available in table S1.  VE against confirmed **HZ-related mortality and hospitalisation** in ZOE-70 MVC (table S2):  Overall: 100 (-9,9-100)  70-79: 100 (-144,1-100)  ≥ 80: 100,0 (-435,1-100,0) |
| Lal H, et al. Efficacy of an adjuvanted herpes zoster subunit vaccine in older adults. N Engl J Med. 2015 May 28;372(22):2087-96.  Multicountry | Phase 3 RCT at 163 sites in 18 countries/regions  1:1 randomized  Placebo-controlled  Triple blind  02.08.10 - 21.07.11  (Same sites and time as ZOE-70)  Funded by industry (GSK)  **FU:** 3,2 years | **Inclusion criteria TVC:**  Age ≥ 50 years  **Exclusion criteria TVC:**  Immunosuppressive condition  History of HZ  HZ or varicella vaccine  Life expectancy < 4 years  **Additional exclusion criteria for the MVC:**  HZ diagnosis ≤ 30 days after dose 2, only 1 dose of RZV. | **Total**:  TVC: 15 411 (50 945* PY)  MVC: 14 759 (46 468* PY)  **Vaccinated**:  TVC: 7 698 (25 585 PY)  MVC: 7 344 (23 297 PY)  **Placebo**:  TVC: 7 713 (25 360 PY)  MVC: 7 415 (23 171 PY) | **Total**:  62,3 ± 9,0 years  **Vaccinated**:62,4 ± 9,0 years  **Placebo**:  62,3 ± 9,0 years | 2 doses RZV I.M vs placebo | **Primary outcome:** Efficacy against HZ ≥ 50 years  **Secondary outcome:**  Efficacy against HZ by age groups  Efficacy in the prevention of overall PHN by age groups  Efficacy in reducing the total duration of severe ‘worst’ HZ-associated pain  Efficacy in the reduction of overall and HZ-related mortality and hospitalizations  Efficacy in the reduction in incidence of HZ-associated complications  Efficacy in the reduction in use of pain medications  Safety  **Exploratory outcomes:**  Efficacy in reducing the severity of acute HZ-associated pain  Efficacy in improving Quality of Life  Efficacy in the mitigation of burden of illness caused by HZ  Evaluate vaccine induced humoral immune responses and anti-VZV neutralizing antibody titers | Not applicable | Overall VE% (95% CI) against **HZ** in ZOE-50 MVC (table 2): 97,2 (93,7-99,0)  VE (95% CI) against **HZ by age-group** in ZOE-50 MVC, years (table 2):  50-59: 96,6 (89,6-99,3)  60-69: 97,4 (90,1-99,7)  ≥ 70: 97,9 (87,9-100,0)  VE against HZ overall and by age group in ZOE-50 TVC available in table 2. |
| López-Fauqued M, et al. Safety profile of the adjuvanted recombinant zoster vaccine: Pooled analysis of two large, randomised phase 3 trials. Vaccine. 2019 Apr 24;37(18):2482-2493. doi: 10.1016/j.vaccine.2019.03.043.  Multicountry | Pooled ZOE-50/70  Post-hoc analysis of ZOE-50 and ZOE-70 to evaluate safety  Funded by industry (GSK)  **FU**: 4,4 years | **Inclusion criteria Pooled vaccinated cohort ≥ 50 years (“pooled ZOE-50/70”) TVC:** Participants in ZOE-50 ≥ 50 years in addition to ZOE-70 participants reporting at least 1 of 15 selected medical conditions at enrolment.  **Exclusion criteria TVC:**  See ZOE-50 and ZOE-70, none of the 15 selcted medical conditions | **Total:**  29 305  **Vaccinated:**  14 645  **Unvaccinated:**  14 660 | Not specified | 2 doses RZV I.M vs placebo | Safety, including events of myocardial infarction (MI) | Not applicable | Myocardial infarction:  RZV-group: 39/14645  Placebo: 39/14660  Acute Myocardial infarction  RZV-group: 13/14645  Placebo: 24/14660  Total:  RR 0.83 (0.57-1.20) |
| Oostvogels L, et al. Medical conditions at enrollment do not impact efficacy and safety of the adjuvanted recombinant zoster vaccine: a pooled post-hoc analysis of two parallel randomized trials. Hum Vaccin Immunother. 2019;15(12):2865-2872.  Multicountry | Pooled ZOE-50/70  Post-hoc analysis of ZOE-50 and ZOE-70 to evaluate RZV efficacy against the first or only episode of HZ and to examine RZV safety in participants with selected medical conditions at enrollment.  Funded by industry (GSK)  **FU**: Not specified | **Inclusion criteria Pooled vaccinated cohort ≥ 50 years (“pooled ZOE-50/70”) TVC:** Participants in ZOE-50 ≥ 50 years in addition to ZOE-70 participants reporting at least 1 of 15 selected medical conditions at enrolment.  **Exclusion criteria TVC:**  See ZOE-50 and ZOE-70, none of the 15 selcted medical conditions  **Additional exclusion criteria for the MVC:**  HZ diagnosis ≤ 30 days after dose 2, only 1 dose of RZV. | **Total:**  MVC: 23 035  (82,5 % of the 27 846* persons in the pooled ZOE-50/70 MVC)  **Vaccinated:**  MVC: 11 427  (82,3% of the 13 881 persons in the pooled ZOE-50/70 MVC)  **Placebo:**  MVC: 11 608  (82,7% of the 14 035 persons in the pooled ZOE-50/70 MVC)  No data on TVC or PY in different cohorts. | **Vaccinated MVC pooled ZOE-50/70**: 68,5 ± 9,8 years  **Placebo MVC pooled ZOE-50/70:**  68,5 ± 9,8 years | 2 doses RZV I.M vs placebo | Efficacy against HZ according to number and type of selected medical conditions at enrolment  Safety | Not applicable | VE% (95% CI) against **HZ by number of selected medical conditions present at enrollment** for pooled ZOE-50/70 MVC (table 2):  1: 95,4 (89,0-98,5)  2: 92,8 (84,7-97,2)  3: 90,5 (80.5-96,0)  ≥3: 90,5 (84,8-94,4)  ≥4: 90,6 (82,4-95,4)  ≥5: 91,2 (78,0-97,3)  ≥6: 90,9 (62,5-99,0) |
| Proano D.A.E, et al. Efficacy, reactogenicity, and safety of the adjuvanted recombinant zoster vaccine for the prevention of herpes zoster in Chinese adults ≥ 50 years: A randomized, placebo-controlled trial. Hum Vaccin Immunother. 2024 Dec 31;20(1):2351584  China | Phase IV observer-blind at 6 centers    05.21 - 04.23    Funded by industry (GSK)  **FU:** 15,2 (±1,1) months | **Inclusion criteria TVC:**  Age ≥ 50 years    **Exclusion criteria TVC:**  Immunsuprressive condition  History of HZ  HZ or varicella vaccine  Unstable comorbidities    **Additional exclusion criteria for the MVC/mES(modified exposed set):**  HZ diagnosis ≤ 30 days after dose 2, only 1 dose of RZV. | **Total:**  TVC: 6138  MVC: 5956    **Vaccinated**  TVC:  MVC: 2965    **Unvaccinated**  TVC:  MVC: 2991 | 62,4 ±7,8 years | 2 doses RZV I.M vs placebo | **Primary outcome:**  Efficacy against HZ    **Secondary outcomes:**  Efficacy by age group    Safety |  | Overall VE% (95% CI) against **HZ** overall, MVC (table 2): 100 (89,8-100). Mean follow up 18 month) (per protocol)  VE% (95% CI) against **HZ 95 (81-99) (**mean follow up 18 months).    VE (95% CI) against **HZ by age-group,** MVC, years (table 2):  50-69: 100 (89,3-100)  ≥ 70: 100 (60,3-100)    Solicited adverse events (AEs) were more frequent in the RZV vs the placebo group (median duration: 1–3 days for both groups). Pain and fatigue were the most frequent local and general AEs (RZV: 72.1% and 43.4%; placebo: 9.2% and 5.3%). The frequencies of unsolicited AEs, serious AEs, potential immune-mediated diseases, and deaths were similar between both groups. RZV is well tolerated |
| Strezova A, et al. Final analysis of the ZOE-LTFU trial to 11 years post-vaccination: efficacy of the adjuvanted recombinant zoster vaccine against herpes zoster and related complications. EClinicalMedicine. 2025 May 9;83:103241.  Multicountry  Publication following:  Strezova A, Diez-Domingo J, Al Shawafi K, Tinoco JC, Shi M, Pirrotta P, Mwakingwe-Omari A; Zoster-049 Study Group. Long-term Protection Against Herpes Zoster by the Adjuvanted Recombinant Zoster Vaccine: Interim Efficacy, Immunogenicity, and Safety Results up to 10 Years After Initial Vaccination. Open Forum Infect Dis. 2022 Oct 23;9(10):ofac485.  and  Boutry C, et al. The Adjuvanted Recombinant Zoster Vaccine Confers Long-Term Protection Against Herpes Zoster: Interim Results of an Extension Study of the Pivotal Phase 3 Clinical Trials ZOE-50 and ZOE-70. Clin Infect Dis. 2022 Apr 28;74(8):1459-1467 | Phase 3 b open label Extension ZOE-50 and ZOE- 70  16.04.16 -juni 2023  Funded by industry (GSK)  **FU:** Up to 12 years, mean ~5,6-11,4 (±0,3) years | **Inclusion criteria TVC:**  At least 1 dose of RZV in ZOE-50 (≥ 50 years) or ZOE-70 (≥ 70 years) and choosing to enrol for the long-term efficacy assessment. Only the vaccinated cohort were offered inclusion in the long-term follow-up.  **Exclusion criteria TVC:**  See ZOE-50 and ZOE-70  **Additional exclusion criteria for the MVC:**  HZ diagnosis ≤ 30 days after dose 2, only 1 dose of RZV. | **Total:**  Not relevant because of historical controls  **Vaccinated:**  TVC: 7 408  (50,6 % of the 14 648 persons in the pooled ZOE-50/70 TVC)  MVC: 7 273 eller 7 258 (39 109,3 PY)  **Placebo (historical control)**:  7 258 (39 109,3 PY) | 67,3 years at first vaccination, 72,5 years at the start of LTFU | 2 doses RZV I.M vs placebo group in ZOE-50/70 year 1-4  2 doses RZV I.M vs historic controls year 6-10 | **Primary outcome:**  Efficacy against HZ from 6 to 12 years after vaccination.  **Secondary outcomes:**  Efficacy against HZ up to 12 years after vaccination  Efficacy against HZ up to 12 years after vaccination by age group  Efficacy against PHN up to 11 years after vaccination  Efficacy against PHN up to 11 years after vaccination by age group  Efficacy against other complications of HZ up to 11 years after vaccination  Efficacy against other complications of HZ up to 11 years after vaccination by age group  Safety | Not applicable | Overall VE% (95% CI) against **HZ** in pooled ZOE-50/70 MVC (table 2 and 3):  1 month- 11 years since vaccination:  87,7 (84,9-90,1)  5-11 years since vaccination:  79,8 (73,7-84,6)  VE% (95% CI) against **HZ by years since vaccination** in pooled ZOE-50/70 MVC (figure 3):  6 years: 83,9  7 years: 83,6  8 years: 82,8  9 years: 73,7  10 years: 71,7  11 years 82,0  VE (95% CI) against **HZ by age-group up to 11 years after vaccination** in pooled ZOE 50/70 MVC, years (table 3):  50-59: 91,7 (86,3-95,4)  60-69: 92,6 (86,7-96,2)  70-79: 83,5 (78,3-87,7)  ≥ 80: 86,8 (76,4-93,2)  Overall VE (95% CI) against **PHN up to 11 years after vaccination** in pooled ZOE-50/70 MVC (table 4):  87,5 (64,8-96,8)  VE (95% CI) against **PHN up to 11 years after vaccination by age-group** in pooled ZOE 50/70 MVC, years (table 4):  50-59: 100 (46,6-100)  60-69: 50 (-860,5-99,2)  70-79: 87,0 (56,8-98,9)  ≥ 80: 50 (-860,5-99,2)  Overall VE (95% CI) against **other complications up to 11 years after vaccination** in pooled ZOE-50/70 MVC (table 4):  91,7 (43,7-99,8)  VE (95% CI) against **other complications up to 11 years after vaccination by age-group** in pooled ZOE 50/70 MVC, years (table 4):  50-59: Undefined  60-69: 100 (-247,2-100)  70-79: 83,3 (-37,4-99,6)  ≥ 80:100 (-247,2-100)  No RZV-related serious adverse events occurred |

| OBSERVATIONAL STUDIES RZV (VE = Vaccine effectiveness) | | | | | | | | |
| --- | --- | --- | --- | --- | --- | --- | --- | --- |
| Florea A, et al. Real-world effectiveness of recombinant zoster vaccine in self-identified Chinese individuals aged ≥50 years in the United States. Hum Vaccin Immunother. 2024 Dec 31;20(1):2327145.  USA | Prospective matched cohort study (KPSC-data)  01.06.18-30.06.22  Funded by industry  **FU total:**  2,09 (±1,01) years  **FU vaccinated:**  2,54 (±0,80) years  **FU unvaccinated:**  1,93 (±1,03) years | **Inclusion criteria:**  Age ≥ 50 years, self-reported chinese ethnicity or self-reportes chinese as prefered language, continuous enrolment 12 months  **Exclusion criteria:**  Second RZV dose < 4 weeks after first dose, HZ-diagnosis < 6 months prior to index date (date of sedond dose) or < 30 days after index date.  Index date= date of 2^nd^ dose | **Total:**  19 890  **Vaccinated:**  3978  **Unvaccinated:**  15 912 | **Total:**  67 (61-73) years  **Vaccinated:**  68 (61-73) years  **Unvaccinated:**  67 (61-73) years | 2 doses RZV I.M vs unvaccinated (interval > 4 weeks and less than 6 months) | **Primary outcome:**  Effectiveness against HZ  **Secondary outcomes:**  Effectiveness against PHN | Matched (1:4) based on age (10-years interval, gender, healthcare utilization characteristics, comorbidities, immunocompromised status, history of ZVL and HZ, continous membership, concomitant vaccination. | Overall adjusted VE% (95% CI) against **HZ (**table 2):  87,6 (78,9-92,7)  Adjusted VE% (95% CI) against **PHN** (table 2):NA  Number of PHN events in vaccine group 0, number of PHN events in unvaccinated group 19. |
| Harris K, et al. The Impact of Routine Vaccinations on Alzheimer's Disease Risk in Persons 65 Years and Older: A Claims-Based Cohort Study using Propensity Score Matching. J Alzheimers Dis. 2023;95(2):703-718.  USA | Retrospective cohort study (OCDM-data)  01.09.11-31.08.19 (ZVL-analysis)  01.09.17 - 31.08.19 (RZV-analysis)  Funding?  **FU fully vaccinated RZV**:  10 months  **FU unvaccinated:**  23 months | **Inclusion criteria:**  Age ≥ 65 years, at least one record between 01.09.09-31.08.17, at least two records during the follow-up periode, at least one dose of RZV (or ZVL)  **Exclusion criteria:**  Diagnosis of dementia, mild cognitive impairment or encehalopathy, prescription of medication aganinst dementia before index date (vaccination date), unknown sex/geographic region/etniciity | **Total:**  397 694  **Vaccinated ≥ 1 dose RZV or ZVL:**  198 847  **Unvaccinated:**  198 847 | **Vaccinated ≥ dose RZV or ZVL:**  72,2 (5,1) years  **Unvaccinated:** 72,1 (5,3)  years | **Primary outcome:**  1 dose of ZVL or RZV vs unvaccinated  **Secondary outcome:**  2 doses RZV I.M vs unvaccinated | **Primary outcome:** Effectiveness against dementia for any HZ-vaccine  **Secondary outcome:**  Effectiveness against dementia for RZV | Matched (1:1) based on age, gender, ethnicity, region, prior ZVL, healthcare utilization, comorbidities, medications, vaccination records.  Used propensity score matching (PSM). | Adjusted (ATT) RR (95% CI) of **dementia for RZV/ZVL** (table 3):  0,75 (0,74-0,77)  Number needed to vaccinate for dementia 37  Adjusted RR (ATT) (95% CI) against **dementia** **for RZV** (table 3):  0,23 (0,21-0,26)  Number needed to vaccinate for dementia 26  Number needed to vaccinate for dementia for other vaccines (treat)  Tetanus, Diphteria, Pertussis NNT 33  Pneumococcal vaccine NNT 34 |
| Helm MF, et al. Recombinant Zoster Vaccine Reduces 3-Year Cardiovascular Risk: Insights From a Multi-Centered Database. J Drugs Dermatol. 2023 Dec 1;22(12):1178-1182  USA | Retrospective matched cohort study (TriNetX)  20.10.17-01.07.19  Not funded by industry  **FU**: 3 years | **Inclusion criteria:**  Age 50-65 years at index  **Exclusion criteria:**  Prior history of stroke, myocardial infarction, mortality or covid-19 infection | **Total:**  15 314 matched in RZV-analysis and 7 222 905 total  **Vaccinated:**  7 657  **Unvaccinated:**  7 657 | **Vaccinated:** 56,33 ±3,62 years  **Unvaccinated:** 56,31 ±3,62 years | 2 doses RZV I.M vs unvaccinated | Effeciveness against stroke, myocardial infarction and/or mortality. | Matched (1:1) based on age, gender, ethnicity, BMI, comorbidities.  Used propensity score matching (PSM). | Adjusted RR (95% CI) of **myocardial infarction,** 3 years: 0,77 (0,55-0,96)  Adjusted RR (95% CI) of **mortality,** 3 years: 0,7 (0,57-0,88)  Adjusted RR (95% CI) of **stroke,** 3 years: 0,97 (0,75-1,26) |
| Izurieta HS, et al. Recombinant Zoster Vaccine (Shingrix): Real-World Effectiveness in the First 2 Years Post-Licensure. Clin Infect Dis. 2021 Sep 15;73(6):941-948.  USA | Prospective cohort study (Medicare data)  01.11.17 - 20.10.19  Not funded by industry  **FU dose 2:** Median 7,1 months  **FU dose 1:** Median 2,9 months | **Inclusion criteria:**  Age ≥ 65 years, continuous enrolment 12 months  **Exclusion criteria:**  HZ diagnosis 1 year prior to index date, not in nursing home/skilled nursing facility or hospice  **Cohorts:**  Switch from unvaccinated to the subsequent vaccinated cohort 30 days after vaccination (first or second dose). | **Vaccinated dose 2:**  1 006 446  (608 928 PY)  **Vaccinated dose 1:**  1 498 275  (478 532 PY)  **Unvaccinated:**  15 589 546  (25 026 129 PY) | **Dose 2:** 74,0 years ±5,9  **Dose 1:** 73,8 years ±5,9  **Unvaccinated:** 74,6 years ±6,7 | 1 dose RZV I.M vs unvaccinated  2 doses RZV I.M vs unvaccinated (interval > 6 months included)  Comparing  - 1 vs 2 doses  - timing of dose 2  - ZVL vaccination status  - immune status | **Primary outcome:**  Effectiveness against HZ  **Secondary outcomes:**  Effectiveness against PHN  Effectiveness against other HZ-related complications: HZO | Age, gender, ethnicity, low-income subsidy status, healthcare utilization characteristics, frailty characteristics, functional immunocompromising chronic conditions, immunocompromising drugs.  Used inverse probability of treatment weighting (IPTW). | Overall adjusted VE% (95% CI) against **HZ (**table 1):  70,1 (68,6-71,5)  Adjusted VE% (95% CI) against **HZ by age-group**, years:  65-79: 70,6 (68,9-72,1)  ≥ 80: 68,5 (65,1-71,6)  Adjusted VE% (95% CI) against **PHN** (table 3):  76,0 (68,4-81,8)  Adjusted VE% (95% CI) against **HZO** (table 3):  66,8 (60,7-72,0)  Adjusted VE against **HZ when history of ZVL prior 5 years** available in table 1.  Adjusted VE against **HZ for immunocompromised individuals** available in table 1. |
| Lu A, et al. Effectiveness of the Recombinant Zoster Vaccine for Herpes Zoster Ophthalmicus in the United States. Ophthalmology. 2021 Dec;128(12):1699-1707.  USA | Retrospective cohort study (OLDW)  01.01.18-31.12.19  Not funded by industry  **FU** median **post-vaccination**: 211 days (IQR, 86-391)  **FU vaccinated** Median 730 days (IQR: 730-730 days)  **FU unvaccinated:** Median 730 days  (IQR: 365 | **Inclusion criteria:**  Age ≥ 50 years, continuous enrolment 12 months  **Exclusion criteria:**  Immunocompromised, HZ diagnosis 1 year prior to index date, RZV prior to index date, only received a single dose of RZV, HZ diagnosis between RZV dose 1 and 2 or ≤ 30 days after dose 2, RZV dose 2 < 30 days or > 210 days after the first dose.  **Cohorts:**  Individuals contributed to unvaccinated person-time until they received the second valid dose RCV, after which they contribute to vaccinated person-time. | **Total:**  4 842 579  (7 491 570* PY)  **Vaccinated:**  177 289  (117 517 PY)  **Unvaccinated:**  4 665 290  (7 374 053 PY) | **Overall median:** 65 years (IQR 56-73)  **Vaccinated median:** 72 years (IQR 69-77)  **Unvaccinated median:** 64 years (IQR 56-73) | 2 doses RZV I.M with an interval of 30-210 days vs unvaccinated | Effectiveness against other HZ-related complications: HZO | Age, gender, ethnicity, region, prior ZVL, antiviral use, healthcare utilization, insurance type, Charlson Comorbidity index  Used inverse probability of treatment weighting (IPTW). | Overall adjusted VE (95% CI) against **HZO** (figure 2): 89,1 (82,9-93,0)  Adjusted VE (95% CI) against **HZO by age-group**, years:  50-59: 100  60-69: 87,6 (59,9-96,2)  70-79: 88,9 (80,6-93,7)  ≥ 80: 88,8 (77,5-94,5)  Adjusted VE against **HZO when history of ZVL less than 1 year prior** available in figure 2. |
| Nelson MR, et al. Active Postlicensure Safety Surveillance for Recombinant Zoster Vaccine Using Electronic Health Record Data  USA | NRSI (prospective cohort, VSD-data) Kaiser Permanente  January 2018 – December 2019  Not funded by industry  **FU:** 42 days | **Inclusion criteria:**  ≥ 50 years, continuous enrollment 12 months  **Comparator cohorts:**  Well-visit comparators had not received RZV, had received influenza vaccine less than 1 year prior to teir well-person health-care visit (=index date).  Historical ZVL-comparators: aged ≥ 60 years who received ZVL from January 2013-December 2017. | **Total:**  1 733 567  **Vaccinated:**  647 307  **Well-visit-comparators:**  1 086 260 | No information | ≥1 dose RZV I.M | Primary outcome:  Safety  Effectiveness against stroke  Effectiveness against myocardial infarction (MI) | Age, gender, region, comorbidity, health-seeking behaviour, prior ZVL, concomitant vaccinations.  Used PSM. | RR (95% CI) of **myocardial infarction** compared to historical ZVL: 0,84  RR (95% CI) of **myocardial infarction** compared to well-visit: 0, 82 (0,72-0,94)  RR (95% CI) of **stroke** compared to historical ZVL: 0,76  RR (95% CI) of **stroke** compared to well-visit: 0,92 (0,79-1,07) |
| Parameswaran GI, et al. Increased Myocardial Infarction Risk Following Herpes Zoster Infection. Open Forum Infect Dis. 2023 Mar 25;10(4):ofad137.  Parameswaran GI, et al. Increased Stroke Risk Following Herpes Zoster Infection and Protection With Zoster Vaccine. Clin Infect Dis. 2023 Feb 8;76(3):e1335-e1340.  USA | Retrospective case-control study (CDW)  January 2010- January 2020 (MI)  January 2010- January 2020 (stroke)  Not funded by industry  **FU:** 90 days | **Inclusion criteria:**  Age ≥ 18 years, primary care visit during the study period  Cases: history of HZ, index date = date of first recorded onset of HZ  Controls: no history of HZ in same period, index date = date of primary care appointment    **Exclusion criteria:**  N/A | **Total/with RZV:**  2 165 584/126 908 (MI)  2 165 505/127 703 (stroke)  **Cases (HZ) total/with RZV:**  71 912/ 1 597 (MI)  71 911/1 592 (stroke)  **Controls (no HZ) total/with RZV:**  2 093 672/126 311 (MI)  2 093 594/126 111 (stroke) | **Cases:** 71 ± 12,4 years  **Controls:**  68,8 ± 13,2 years | ≥1 dose RZV I.M | Effectiveness against stroke within 30 days  Effectiveness against myocardial infarction (MI) within 30 days | Age, gender, ethnicity, prior HZ, comorbidity.  Used multivariable backwards elimination, comparable to propensity score matching (PSM). | OR (95% CI) of **stroke** within **30 days** postevent: 0,57 (0,46-0,72)  OR (95% CI) of **MI** within **30 days** postevent in group ≥ 18 years (exactly the same as in the group ≥ 50 years, supplementary table 1) : 0,82 (0,74-0,92) |
| Polisky V, et al. Varicella-zoster virus reactivation and the risk of dementia. Nat Med. 2025 Dec;31(12):4172-4179.  USA  Following:  Schwab P. et al, 2024,  https://doi.org/10.1002/alz.088064 | Retrospective  matched cohort (OLDW)    01.10.07-20.09.23    Funded by industry  **FU**: Not specified | **Inclusion criteria RZV-cohort:**  Age ≥ 50 years, vaccination after RZV-approval (20.10.17), ≤ 3 doses of RZV  **Inclusion criteria ZVL-cohort:**  Age ≥ 50 years, vaccination after ZVL-approval (25.05.06), ≤ 3 doses of ZVL  **Inclusion criteria PPSV23-cohort:**  Age ≥ 50 years, vaccination after PPSV23-approval (30.06.83), ≤ 3 doses of PPSV23, no dementia diagnosis within 10 days after vaccination  Individuals without a specific cohort-defining event were included in the respective not-exposed cohorts. The not-exposed cohort for comparisons against individuals vaccinated with either RZV or ZVL only included people who did not receive either of the two HZ vaccines but could receive PPSV23.    **Exclusion criteria:**  Not documented birth year, invalid death status, index date before birth or after death, index date before 01.10.07, fewer than 25 recorded encounters, dementia-diagnosis before index date, did not have an index date.  PPSV23 following after RZV (when comparing RZV 2+ doses vs PPSV23), PPSV23 following less than 1 year after RZV (when comparing RZV vs PPSV23), ZVL following less than 30 days after RZV in the RZV 1 dose cohort (when comparing RZV 2+ dose with RZV 1 dose), ZVL following less than 1 year after ZVL (when comparing RZV 2+ doses vs ZVL).  For comparisons between vaccinated individuals, we also excluded individuals from one cohort who received the vaccine from the other cohort before the index date. | Total/not censored:  112 640/18 056^1^  468 618/16 152^2^  833 530/29 074^3^  Vaccinated RZV total/not censored:  56 320/9 153^1^  234 309/9 031^2^  416 765/16 756^3^  Control total/not censored:  56 320/8 903^1^ (ZVL)  234 309/7 121^2^ (PPSV23)  416 765/12 318^3^ (RZV 1 dose)  ^1^ RZV (2+ doses) vs ZVL  ^2^ RZV (2+ doses) vs PPSV23  ^3^ RZV (2+ doses) vs RZV (1 dose) | RZV-group:  65,0 years^1^  65,6 years^2^  65,1 years^3^  Comparator:  64,8 years (ZVL)^1^  65,2 years (PPSV23)^2^  65,1 years (RZV 1 dose)^3^  ^1^ RZV (2+ doses) vs ZVL  ^2^ RZV (2+ doses) vs PPSV23  ^3^ RZV (2+ doses) vs RZV (1 dose) | ≥2 doses RZV I.M vs ≥1 dose ZVL  ≥2 doses RZV I.M vs ≥1 dose PPSV23  ≥2 doses RZV I.M vs ≥1 dose RZV | Effectiveness against dementia | Nearly 400 pre-exposure characteristics, including demographics, comorbidities/lifestyle factors (for example, smoking status, cardiovascular conditions and diabetes), healthcare utilization and healthcare-seeking behaviors.  Used PSM. | RR (95% CI) of **dementia within 3 years since vaccination compared to ZVL**: 0,89 (0,74-1,1)  RR (95% CI) of **dementia within 5 years since vaccination compared to ZVL**: 0,83 (0,69-0,98)  RR (95% CI) of **dementia within 3 years since vaccination compared to PPSV23**: 0,73 (0,66-0,80)  RR (95% CI) of **dementia within 5 years since vaccination compared to PPSV23**: 0,83 (0,74-0,94)  RR (95% CI) of **dementia within 3 years since vaccination compared to 1 dose RZV**: 0,76 (0,71-0,82)  RR (95% CI) of **dementia within 5 years since vaccination compared to 1 dose RZV**: 0,81 (0,74-0,88) |
| Sun Y, Jackson K, et al. Effectiveness of the recombinant zoster vaccine among Kaiser Permanente Hawaii enrollees aged 50 and older: A retrospective cohort study. Vaccine. 2021 Jun 29;39(29):3974-3982.  USA | Retrospective cohort study (KPH)  01.01.18 - 31.12.19  Not funded by industry  **FU:**  Both cohorts: Median 730 days (IQR 480-730)  IQR **vaccinated** cohort: 730-730 days  IQR **unvaccinated** cohort: 430-730 days | **Inclusion criteria:**  Age ≥ 50 years, continuous enrolment 12 months  **Exclusion criteria:**  Immunocompromised, HZ diagnosis 1 year prior to index date, only received a single dose of RZV, RZV dose 1 before index date or before 01.01.18, HZ diagnosis between RZV dose 1 and 2 or ≤ 30 days after dose 2, joined KPH after 01.01.18, RZV dose 2 < 30 days or > 210 days after the first dose.  **Cohorts:**  Individuals contributed to unvaccinated person-time until they received the second valid dose RZV, after which they contribute to vaccinated person-time. | **Total:**  78 356  (128 010* PY)  **Vaccinated:**  11 864  (8 291 PY)  **Unvaccinated:**  66 492  (119 719 PY) | **Overall median:** 61 years (IQR 54-69)  **Vaccinated median:** 74 years (IQR 70-80)  **Unvaccinated median:** 59 years (IQR 53-65) | 2 doses RZV I.M with an interval of 30-210 days vs unvaccinated | Effectiveness against HZ  Effectiveness against HZ by age groups  Effectiveness against other HZ-related complications: HZO | Age, gender, ethnicity, region, prior ZVL, antiviral use, healthcare utilization, insurance type, Charlson Comorbidity index.  Used inverse probability of treatment weighting (IPTW). | Overall adjusted VE (95% CI) against **HZ** (table 4):  83,5 (74,9-89,2)  Adjusted VE (95% CI) against **HZ** **by age-group,** years (table 4):  50-59: 100  60-69: 67,7 (11,8-88,1)  70-79: 83,3 (70,1-90,7)  ≥ 80: 86,4 (73,5-93,0)  Overall adjusted VE (95% CI) against **HZO** (text):  93,3 (48,7-99,1)  Adjusted VE against **HZ when history of prior ZVL** (table 4):  61,1 (-124,9-93,3) |
| Sun Y, Kim E, et al. Effectiveness of the Recombinant Zoster Vaccine in Adults Aged 50 and Older in the United States: A Claims-Based Cohort Study. Clin Infect Dis. 2021 Sep 15;73(6):949-956.  USA | Retrospective cohort study (OLDW)  01.01.18-31.12.19  Not funded by industry  **FU:**  Median post-vaccination: 7,0 months (IQR 2,8-13,0 months) | **Inclusion criteria:**  Age ≥ 50 years, continuous enrolment 12 months  **Exclusion criteria:**  Immunocompromised, HZ diagnosis 1 year prior to index date, RZV prior to index date, only received a single dose of RZV, HZ diagnosis between RZV dose 1 and 2 or ≤ 30 days after dose 2, RZV dose 2 < 30 days or > 210 days after the first dose.  **Cohorts:**  Individuals contributed to unvaccinated person-time until they received the second valid dose RCV, after which they contributed to vaccinated person-time. | **Total:**  4 769 819  (7 300 036* PY)  **Vaccinated:**  173 745  (115 125 PY)  **Unvaccinated:**  4 596 074  (7 184 911 PY) | **Overall median:** 65 years (IQR 56-73)  **Vaccinated median:** 72 years (IQR 69-77)  **Unvaccinated median:** 64 years (IQR 56-73) | 2 doses RZV I.M with an interval of 30-210 days vs unvaccinated | Effectiveness against HZ | Age, gender, ethnicity, region, prior ZVL, antiviral use, healthcare utilization, insurance type, Charlson Comorbidity index.  Used inverse probability of treatment weighting (IPTW). | Overall adjusted VE (95% CI) against **HZ** (figure 2):  85,5 (83,5-87,3)  Adjusted VE (95% CI) against **HZ by age-group**, years (figure 2):  50-59: 85,6(53,3-95,6)  60-69: 87,7 (82,5-91,4)  70-79: 86,5 (83,9-88,6)  ≥ 80: 80,3 (75,1-84,3)  Adjusted VE against **HZ when history of ZVL less than 5 years prior** available in text. |
| Tang E, et al. Recombinant zoster vaccine and the risk of dementia. Vaccine. 2025 Feb 6;46:126673. doi: 10.1016/j.vaccine.2024.126673. Epub 2024 Dec 28.  USA | Retrospective cohort (OLDW)    01.01.18-31.12.22    Not funded by industry  **FU**: Not specified | **Inclusion criteria:**  Age ≥ 50 years, continuous enrolment 12 months    **Exclusion criteria:**  Prior dementia diagnosis, immunocompromised status, RZV prior to 50 years of age or index date    **Cohorts:**  Individuals contributed to unvaccinated person-time until 29 days after first dose, partially vaccinated person-time from 30 days after first dose until 29 days after second dose, and fully vaccinated person-time from 30 days after second dose.  Index date = date an individual was eligible for study inclusion | **Total**:  4 502 678  (12 247 881 PY)    **Fully vaccinated:**  460 413  (826 733 PY)    **Partially vaccinated:**  260 297  (392 353 PY)    **Unvaccinated:**  3 835 968  (11 028 795 PY) | **Total**:  62 (54-71)    **Fully vaccinated, at index date:**  67 (58-73)  Median age at dose 2; 69 years IQR (60-75)  **Partially vaccinated, at index date:**  63 (54-71)  Median age at dose 1: 67 years (IQR 59-74)    **Unvaccinated:**  62 (53-71) | ≥1 dose RZV I.M vs unvaccinated  Comparing  - 1 vs 2 doses  - age groups  - gender  - HZ-diagnosis before or after vaccination  -use of anitvirals | Effectiveness against dementia | Age, gender, ethnicity, region, prior ZVL last year, HZ-diagnosis, RZV-vaccination, antivirals, Charlson Comorbidity Index.  Used time-varying Cox proportional haszards model accounting for time-fixed and time-varying covariates. | Adjusted HR of **dementia** (95% CI) (table 3):  1 dose: 0,89 (0,87-0,92)  2 doses: 0,68 (0,67-0,7)    Herpes zoster diagnosis before the first vaccination dose for dementia  aHR 1.47 (1.42-1.52)  Additional information in table 3:  HZ diagnosis, regardless of receipt or timing of RZV increased risk of dementia.    Antivirals reduced risk of dementia.    Risk of dementia increased with age groups and was higher for women. |
| Taquet M, et al. The recombinant shingles vaccine is associated with lower risk of dementia. Nat Med. 2024 Oct;30(10):2777-2781.  USA | Retrospective cohort study (TriNetX)  RZV-cohort: 01.10.14-30.10.17  FU: 4,15 (3,16-4,99) years  Comparator cohort: 01.11.17-31.10.20  FU: 6,0 (5,2-6,0) years  Not funded by industry, author has bonds to GSK | **Inclusion criteria:**  Age ≥ 65 years when receiving first HZ-vaccine  **Exclusion criteria:**  Dementia-related diagnosis  **Cohorts:**  Individuals who received their first dose of HZ-vaccine (95% RZV) between November 2017 – October 2020 was included in the RZV-cohort.  Individuals who received their first dose of HZ-vaccine (98% ZVL) between October 2014 – September 2017 was included in the comparator (ZVL) cohort. | **Total:**  206 774  **RZV-cohort:**  103 387  **Comparator cohort (ZVL):**  103 387 | **RZV-cohort:**  70,7 ± 4,8 years  **Comparator cohort (ZVL):**  70,7 ± 4,8 years | ≥1 dose RZV I.M vs 1 dose of ZVL I.M or unvaccinated  Natural experiment at time of rapid transition of ZVL to RZV I US | Effectiveness against dementia | Matched (1:1) based on age, gender, ethnicity, marital status, prior HZ-infection, prior influenzae-vaccine,  Comorbidity.  Used propensity score matching (PSM). | Restricted mean time loss (RTML) ratio to **dementia** (95% CI): 0,83 (0,80-0,87)  Translating into 17% time lived diagnosis-free, or 164 (95% CI 124-202) additional diagnosis-free days among those affected. |
| Walia A, et al. Risk of Herpes Zoster Ophthalmicus Recurrence After Recombinant Zoster Vaccination. JAMA Ophthalmol. 2024 Mar 1;142(3):249-256.  USA | Retrospective matched cohort (OLDW)    01.01.10-31.12.22    Not funded by industry  **FU**: Not specified | **Inclusion criteria:**  Prior HZO-diagnosis and age ≥ 50 years, continuous enrolment 12 months    **Exclusion criteria:**  Prior dementia diagnosis, immunocompromised status, RZV prior to 50 years of age or index date    **Cohorts:**  Individuals contributed to vaccinated person-time if they received at least 1 dose of RZV more than 90 days following the initial HZO. | **Total**:  16 408    **Vaccinated:**  5 998 (901 PY)    **Unvaccinated:**  12 762 (1 910 PY) | Age at initial HZO diagnosis    **Vaccinated:** 67,4 (9,8 SD)    **Unvaccinated:**  68,6 (10,3 SD) | ≥1 dose RZV I.M  vs unvaccinated  Comparing  - 1 vs 2 doses | Effectiveness against HZO recurrence | Matching (1:10) based on age at initial HZO, year of intitial HZO-diagnosis, probable ocular involvement at initial HZO episode, time of follow-up on dose-level.  Used time-varying Cox proportional haszards model accounting for time-fixed and time-varying covariates. | Adjusted HR of **recurrent HZO** (95% CI) up to 56 days after vaccination (figure 2):  Total: 1,64 (1,01-2,67)  Dose 1: 1,64 (0,93-2,88)  Dose 2: 1,65 (0,87-3,13) |
| Xu X, et al. Protective Effects of Recombinant Zoster Vaccine and Antiviral Therapy Against Cardiovascular Disease Following Herpes Zoster Infection. J Infect Dis. 2025 Aug 14;232(2):465-473.  USA | Retrospective cohort (OLDW)    01.01.18-31.03.23    Not funded by industry    **FU**: Not specified  Total: 1,9 years  RZV: 1,5 years  Unvaccinated: 2,0 years | **Inclusion criteria:**  Age ≥ 50 years, continuous enrolment 12 months, HZ-diagnose after reaching vaccine age eligiblity.    **Exclusion criteria:**  HZ less than 1 year before index date, CVD-diagnosis less than 1 year before index date, receiving RZV after HZ    **Cohorts:**  Patients were defined as unvaccinated if they did not receive RZV or if they had an HZ infection within 30 days of their first dose of RZV.  Partially vaccinated: were diagnosed with HZ between 30 days after first dose until 29 days after second dose.  Fully vaccinated: diagnosed with HZ ≥30 days after second dose.  Index date: date of first HZ diagnosis after becoming eligible for RZV | **Total:**  112 637  (216 097,8 PY)    **Vaccinated (partially og fully):**  7 884  (11 835,3 PY)    **Unvaccinated:**  104 753  (204 262,5 PY) | **Total:**  70 (59-77)    **Vaccinated** 73 (65-79)    **Unvaccinated:**  69 (59-77) | ≥1 dose RZV I.M vs unvaccinated    Anitviral treatment or not. | Effectivenes against cardiovascular disease event (stroke, myocardial infarction, heart failure, unstable angina).    Effectiveness against all-cause mortality | Age, gender, ethnicity, region, prior ZVL last year, Charlson Comorbidity Index, cardiovascular medications.    Used time-fixed Cox proportional haszards model. | Adjusted HR against **cardiovascular disease event** (95% CI) within **365 days** after HZ-infection (table 3):  RZV only: 0,79 (0,67-0,94)  Both antiviral treatment and RZV: 0,61 (0,53-0,72)    Adjusted HR against **all-cause mortality** (95% CI) within 365 days after HZ-infection (table 3):  RZV only: 0,64 (0,32-1,25)  Both antiviral treatment and RZV: 0,36 (0,18-0,69) |
| Zerbo O, et al. Effectiveness of Recombinant Zoster Vaccine Against Herpes Zoster in a Real-World Setting. Ann Intern Med. 2024 Feb;177(2):189-195.  USA | Prospective cohort (4 VSD sites)    01.01.18 – 31.12.22    Not funded by industry  **FU:**  Total:  3,8 years    Vaccinated: 1,6 years    Unvaccinated:  3,2 years | **Inclusion criteria:**  Age ≥ 50 years, continuous enrolment 12 months    **Exclusion criteria:**  Prior RZV, HZ diagnosis 1 year prior to index date.    **Cohorts:**  Individuals contributed to unvaccinated person-time until 29 days after first dose, partially vaccinated person-time from 30 days after first dose until 29 days after second dose, and fully vaccinated person-time from 30 days after second dose.    Subdivided RZV-status based on the inverval since receipt of last dose (30 days to < 1 year, 1 to < 2 years, 2 to < 3 years, or ≥ 3 years).    Subdivided fully vaccinated based on interval between doses (28 days-6 months, > months to < 1 year, and ≥ 1 year) | **Total:**  1 996 885  (>7 600 000 PY)    **Vaccinated dose 2:**  576 483    **Vaccinated dose 1:**  761 042      **Unvaccinated:**  104 753  (204 262,5 PY) | Not specified | ≥1 dose RZV I.M vs unvaccinated | Effectiveness against HZ up to 4 years after vaccination    Effectiveness against HZ by age at first RZV dose | Age, gender, ethnicity, region, prior ZVL, corticosteroid use, influenza vaccination, healthcare utilization, 5 health conditions.  Used Cox proportional haszards model accounting for time-fixed and time-varying covariates. | Adjusted VE% (95% CI) against **HZ (fully vacicnated)** (figure 1):  76 (75-78)    Adjusted VE% (95% CI) against **HZ by years since vaccination** for fully vaccinated (figure 2):  1 year: 79 (77-81)  2 years: 75 (72-77)  3 years: 73 (69-76)  ≥ 3 years: 73 (68-78)    Adjusted VE% (95% CI) against **HZ by age at first RZV dose** (figure 3):  50-64 years: 81 (78-83)  ≥ 65 years: 74 (73-76)    Adjusted VE% (95% CI) against **HZ** (figure 3) by cortocoisteroid use:  No: 77 (76-79)  Yes: 65 (59-70) |
