# Supplementary material for: Efficacy and Effectiveness of the Recombinant Zoster Vaccine Against Herpes Zoster, Herpes Zoster Ophthalmicus, Postherpetic Neuralgia and Dementia: A Systematic Review and Meta‐Analysis
Source: Rev Med Virol. 2026 Sep 16;36(5):e70203. doi: 10.1002/rmv.70203 (PMC13581344; doi:10.1002/rmv.70203)
Supplement: Supplementary file 6 [file RMV-36-e70203-s006.docx]

**Supplementary table S2:** Risk of bias

For RCTs we used version 2 of the Cochrane risk-of-bias tool for randomized trials (RoB 2) and for observational studies version 1 we used the Cochrane Risk Of Bias In Non-randomized Studies of Interventions (ROBINS-I).

Supplementary table S2a. Risk of bias in RCT (RoB 2).

**Supplementary table S2b**. Risk of bias in observational studies (ROBINS-I).
