# Supplementary material for: Efficacy and Effectiveness of the Recombinant Zoster Vaccine Against Herpes Zoster, Herpes Zoster Ophthalmicus, Postherpetic Neuralgia and Dementia: A Systematic Review and Meta‐Analysis
Source: Rev Med Virol. 2026 Sep 16;36(5):e70203. doi: 10.1002/rmv.70203 (PMC13581344; doi:10.1002/rmv.70203)
Supplement: Supplementary file 7 [file RMV-36-e70203-s007.docx]

**Supplementary table S6:** Protective effects of RZV vs unvaccinated against cardiovascular diseases in adults above 50 or 65 years.

|  | **Total number of patients in the study** | **Stroke**  **(95% CI)** | **Myocardial infarction**  **(95% CI)** | **Total cardiovascular diseases** *****  **95% CI)** |
| --- | --- | --- | --- | --- |
| Lopez-Fauqued et al.  (FU 4.4 year) (46) | 29 306 |  | RR 0.83  (0.57-1.20) |  |
| Helm et al. 2023  (FU 36 month, PSM) (52) | 15 314 | aRR 0.97  (0.75-1.26) | aRR 0.73  (0.55-0.96) |  |
| Nelson et al. 2023  (FU 23 month) (55) | 1 733 567 | aRR 0.92  (0.79-1.07) | aRR 0.82  0.72-0.94) |  |
| Parameswaran  et al.  2023ab (FU 30 days) (56,57) | 127 703 | OR 0.57  (0.46-0.71) | OR 0.82  (0.74-0.92) |  |
| Xu et al. 2025  (FU 12 month) (63) | 108 112 |  |  | aHR 0.78  (0.63-0.95) |

PSM; propensity score matching, CI: confidens interval, FU: Follow up, aRR; adusted risik reduction, aHR: jadjusted hazard ratio, OR: odds ratio, *Stroke, Myocardial infarction, heart failure or unstable angina).
